# A Prospectively Validated Prognostic Model for Patients with Locally Advanced Squamous Cell Carcinoma of the Head and Neck Based on Radiomics of Computed Tomography Images

**DOI:** 10.3390/cancers13133271

**Published:** 2021-06-29

**Authors:** Simon A. Keek, Frederik W. R. Wesseling, Henry C. Woodruff, Janita E. van Timmeren, Irene H. Nauta, Thomas K. Hoffmann, Stefano Cavalieri, Giuseppina Calareso, Sergey Primakov, Ralph T. H. Leijenaar, Lisa Licitra, Marco Ravanelli, Kathrin Scheckenbach, Tito Poli, Davide Lanfranco, Marije R. Vergeer, C. René Leemans, Ruud H. Brakenhoff, Frank J. P. Hoebers, Philippe Lambin

**Affiliations:** 1 The D-Lab, Department of Precision Medicine, GROW-School for Oncology, Maastricht University, Maastricht, Universiteitssingel 40, 6229 ER Maastricht, The Netherlands; s.keek@maastrichtuniversity.nl (S.A.K.); h.woodruff@maastrichtuniversity.nl (H.C.W.); S.primakov@maastrichtuniversity.nl (S.P.); 2 Department of Radiation Oncology (MAASTRO), GROW-School for Oncology and Developmental Biology, Maastricht University Medical Centre+, Postbus 3035, 6202 NA Maastricht, The Netherlands; frederik.wesseling@maastro.nl (F.W.R.W.); frank.hoebers@maastro.nl (F.J.P.H.); 3 Department of Radiology and Nuclear Medicine, GROW-School for Oncology, Maastricht University Medical Centre+, P.O. Box 5800, 6202 AZ Maastricht, The Netherlands; 4 Department of Radiation Oncology, University Hospital Zürich, University of Zürich, Rämistrasse 100, 8091 Zürich, Switzerland; Janita.vanTimmeren@usz.ch; 5 Amsterdam UMC, Otolaryngology/Head and Neck Surgery, Cancer Center Amsterdam, Vrije Universiteit Amsterdam, Postbus 7057, 1007 MB Amsterdam, The Netherlands; i.nauta@amsterdamumc.nl (I.H.N.); cr.leemans@amsterdamumc.nl (C.R.L.); rh.brakenhoff@amsterdamumc.nl (R.H.B.); 6 Department of Otorhinolaryngology, Head Neck Surgery, i2SOUL Consortium, University of Ulm, Frauensteige 14a (Haus 18), 89075 Ulm, Germany; t.hoffmann@uniklinik-ulm.de; 7 Head and Neck Medical Oncology Unit, Fondazione IRCCS Istituto Nazionale dei Tumori, via Giacomo Venezian, University of Milan, 1 20133 Milano, Italy; stefano.cavalieri@istitutotumori.mi.it (S.C.); lisa.licitra@istitutotumori.mi.it (L.L.); 8 Radiology Unit, Fondazione IRCCS Istituto Nazionale dei Tumori via Giacomo Venezian, 1 20133 Milano, Italy; giuseppina.calareso@istitutotumori.mi.it; 9 Oncoradiomics SA, Liège, Clos Chanmurly 13, 4000 Liège, Belgium; ralph.leijenaar@oncoradiomics.com; 10 Department of Oncology and Hemato-Oncology, University of Milan, via S. Sofia 9/1, 20122 Milano, Italy; 11 Department of Medicine and Surgery, University of Brescia, Viale Europa, 11-25123 Brescia, Italy; marcoravanelli@hotmail.it; 12 Department. of Otorhinolaryngology-Head and Neck Surgery, University Hospital Düsseldorf, Moorenstr. 5, 40225 Düsseldorf, Germany; Scheckenbach@med.uni-duesseldorf.de; 13 Maxillofacial Surgery Unit, Department of Medicine and Surgery, University of Parma-University Hospital of Parma, via Università, 12-I, 43121 Parma, Italy; tito.poli@unipr.it (T.P.); lanfranco82@yahoo.it (D.L.); 14 Amsterdam UMC, Cancer Center Amsterdam, Department of Radiation Oncology, Vrije Universiteit Amsterdam, Postbus 7057, 1007 MB Amsterdam, The Netherlands; mr.vergeer@amsterdamumc.nl

**Keywords:** radiomics, machine learning, precision medicine, head and neck cancer, survival study

## Abstract

**Simple Summary:**

Patients that suffer from advanced head and neck cancer have a low average survival chance. Improving prognosis could improve this survival rate as it may help in clinical decision making. Radiomics features calculated from images of the tumour describe tumour size, shape, and pattern. These characteristics may be linked to patient survival, which is investigated in this paper. We combined radiomics features with other biomarkers of survival of 809 patients to make a prognosis before treatment. We then compared the predicted prognosis with the actual outcome to see how well our model performs. Our model was able to make three distinct risk groups of low-, medium-, and high-survival patients. With these findings, doctors may make a better judgement of treatment and follow-up per patient, which might improve clinical outcomes.

**Abstract:**

Background: Locoregionally advanced head and neck squamous cell carcinoma (HNSCC) patients have high relapse and mortality rates. Imaging-based decision support may improve outcomes by optimising personalised treatment, and support patient risk stratification. We propose a multifactorial prognostic model including radiomics features to improve risk stratification for advanced HNSCC, compared to TNM eighth edition, the gold standard. Patient and methods: Data of 666 retrospective- and 143 prospective-stage III-IVA/B HNSCC patients were collected. A multivariable Cox proportional-hazards model was trained to predict overall survival (OS) using diagnostic CT-based radiomics features extracted from the primary tumour. Separate analyses were performed using TNM8, tumour volume, clinical and biological variables, and combinations thereof with radiomics features. Patient risk stratification in three groups was assessed through Kaplan–Meier (KM) curves. A log-rank test was performed for significance (*p*-value < 0.05). The prognostic accuracy was reported through the concordance index (CI). Results: A model combining an 11-feature radiomics signature, clinical and biological variables, TNM8, and volume could significantly stratify the validation cohort into three risk groups (*p* < 0∙01, CI of 0.79 as validation). Conclusion: A combination of radiomics features with other predictors can predict OS very accurately for advanced HNSCC patients and improves on the current gold standard of TNM8.

## 1. Introduction

Head and neck squamous cell carcinomas (HNSCC) are cancerous tumours that typically grow in the oral cavity (OC), larynx, and pharynx. In Europe, 140,000 new cases are diagnosed yearly leading to approximately 70,000 deaths [1]. Despite advances in treatment, 3-years overall survival (OS) for locoregionally advanced HNSCC remained 40–50% [2,3,4]. Management of HNSCC patients starts with a diagnostic workup of the tumour, lymph node metastases, and distant metastases (TNM) to stage the tumour. Furthermore, immunostaining determined p16 protein expression, acting as a surrogate marker of HPV infection, has been included as an important factor in the American Joint Committee on Cancer (AJCC) 8th edition for staging of oropharyngeal cancer, which introduced separate staging systems for p16-positive and p16-negative oropharyngeal carcinomas [5]. Besides the TNM stage, prognosis depends on clinical (e.g., patients’ comorbidities, performance status) and biological (e.g., invasive growth or gene expression) factors, and for patients treated with surgery, on microscopic examination of the resection specimen [4]. RNA and DNA profiling have identified molecular subtypes of HNSCC with different prognoses [6]. Some of these subtypes may include primary tumours with high heterogeneity which may react differently to treatment [7]. Defining a robust and clinically viable method to determine these subtypes is therefore essential for the effective treatment of HNSCC patients.

Routine pre-treatment radiological imaging provides a source of non-invasively acquired information of the primary tumor that could be investigated for the ability to determine clinically relevant subtypes. Advanced image analysis methods such as radiomics allow for the analysis of radiographic medical images by extracting large amounts of so-called features using mathematical algorithms and finding correlations with biological and/or clinical outcomes using machine learning techniques. Previous studies have shown that radiomics in computed tomography (CT) imaging can improve the prediction of prognosis of HNSCC [8,9,10,11,12,13,14,15,16,17,18,19,20,21,22,23,24,25,26,27,28,29,30,31]. Radiomics on CT HNSCC imaging has been used to predict HPV status [8,9], overall survival [10,11,12,13,14], progression-free survival [10,12,13,14], local tumour control [8,12,15,16,17,18,19,20,21], tumour grade [9,22], lymph node response [23,24], tumour invasiveness [9,25], xerostomia [26,27,28], tumour resectability [29], and classifying molecular subtypes [30,31]. While the survival studies show that radiomics on CT data can significantly stratify patients in multiple survival groups, performance expressed through Harrell’s C index ranged widely, from 0.58 to 0.9. An explanation of these discrepancies is that radiomics studies are commonly limited in data, with patient numbers for HNSCC regularly using around 100–200 patients combined for training and validation. Furthermore, the data are usually collected from two centres—one for training and one for validation. To create radiomics models which have sufficient predictive power and that are generalisable across different patient populations, large datasets from multiple institutes are needed.

We hypothesise that the multicentric ‘Big Data and Models for Personalised Head and Neck Cancer Decision Support’ project (BD2Decide) [32,33] dataset provides the necessary breadth to create statistically significant, high-quality models that can add complementary information to other well-known but under-utilised clinical and biological factors [34,35,36]. In addition, we hypothesise that the international multicentric nature of the data will, compared to many contemporary HNSCC radiomics-based studies, give us the necessary variation in the dataset to generalise the model across different patient populations. Similar to the inclusion of HPV status to TNM8, we believe that combining these factors may improve the prediction of patient prognosis instead of using them independently. We also hypothesise that a multifactorial machine learning model, including radiomics features derived from the primary tumour, can outperform the current gold standard (TNM8) in stratifying locally advanced HNSCC patients into OS risk groups. This new signature of radiomics features was compared against an existing signature. Furthermore, mixed models containing TNM, tumour volume, radiomics features, clinical variables, and biological variables were developed and validated.

## 2. Materials and Methods

### 2.1. Patient Characteristics

Protocol details were registered on Open Science Framework (DOI number: 10.17605/OSF.IO/H4DFB). The study population was composed of locoregionally advanced HNSCC patients (TNM7 stage III-IVA/B (M0)) receiving curative treatment between 2008 and 2017, collected within the framework of the BD2Decide project (http://www.bd2decide.eu/, accessed on 13 May 2021, H2020-PHC30-689715, IRB P-number P0125, ClinicalTrials.gov Identifier: NCT02832102) [32,33]. The collected patient population was originally staged at diagnosis of the TNM7 staging system. During the BD2Decide project, these patients were re-staged to I-IVA/B (M0) using the newly developed TNM8 staging system. The ethical approval statement and an overview of the inclusion criteria can be found in Supplementary Materials. Patients’ data were collected both retrospectively (diagnosis between 2008 and 2014) and prospectively (diagnosis between 2015 and 2017). The retrospective and the prospective datasets were assigned as the training and validation datasets, respectively. OS was established as the period between the primary diagnosis and the date of death or last follow-up, with at least three years of follow-up performed. Patients alive with less than 2-year follow-up were excluded and defined as ‘lost to follow-up’. Median follow-up times were determined separately for training and validation datasets through the reverse Kaplan–Meier (KM) estimate [37]. The similarity in patient characteristics between cohorts was assessed through two-proportion z-tests to test whether there is a difference in a categorical variable, or unpaired two-sample t-tests to test whether there is a difference in a numerical variable. For the latter, the assumptions of the data having a normal distribution and possessing the same variance in both cohorts were tested through Shapiro–Wilk’s test and f-test, respectively. The significance level was set to 5%.

### 2.2. CT Acquisition Parameters

**Figure 1:**
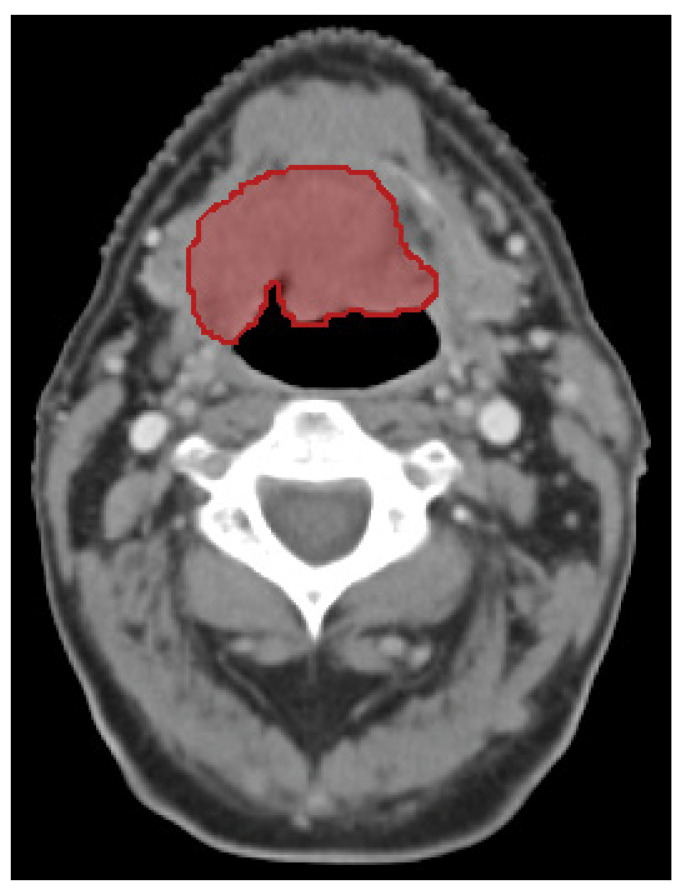
Computed tomography image of patient with stage IVA oropharyngeal cancer in transverse plane. The primary tumour is shown outlined in red.

CT images were acquired at each centre with scanners, acquisition protocols, and reconstruction protocols according to standard operating procedures (SOPs) at the respective centres for diagnostic imaging. All CT images were either diagnostic or radiotherapy treatment planning images of comparable diagnostic quality, all with an intravenous contrast injection protocol. All CT scans within the framework of the BD2Decide project had a 3 mm slice thickness or less. Any CT scan that had imaging artifacts in more than 50% of the slices with primary tumour mass present was excluded. For patients who received radiotherapy, the primary gross tumour volume (GTV) was delineated at each centre according to local delineation guidelines by experienced radiation oncologists. The GTV was defined as the visual extent of gross tumour volume, as described in the radiology report and, if needed, adapted based on the report of the physical examination. Figure 1 gives an example of a CT with the primary tumour delineated. For patients who did not receive radiation treatment, the primary tumour volume was delineated locally by or supervised by expert radiologists according to local delineation protocols. This delineation was conducted by a single person per centre, directly on the contrast-enhanced (CE)-CT. CE-CT has shown to have lower interobserver variability for HNSCC delineation, compared to just CT, or PET-CT [38]. Additionally, for all patients treated with radiotherapy from Maastro, VUmc, and the University of Brescia, all contours were delineated on CT in conjunction with PET/MRI, which has also been proven to greatly decrease interobserver variation [39,40]. All contours were additionally peer-reviewed by radiation oncologists based on diagnostic information. Lastly, all delineations were visually judged by a single observer in the BD2Decide consortium for deficiencies. Supplementary Materials Table S1 provides an overview of the treatment received per participating centre.

### 2.3. Feature Extraction

Radiomics features were obtained from the delineated primary tumour volume of the preprocessed images. A full list of software packages used in the present study is shown in Table S2 of the Supplementary Materials. Feature extraction was performed in python 3.6.10, with the package PyRadiomics version 2.2.0 [41]. To lessen the impact of heterogeneity in the imaging data caused by differences in scanners and imaging protocols, preprocessing of the images and postprocessing of the extracted features were performed. An overview of pre- and postprocessing techniques applied to the data has been described in Supplementary Materials. Both International Biomarker Standardisation Initiative (IBSI)-compliant [42,43] and a non-IBSI compliant feature were extracted. Features extracted through PyRadiomics contain a single first-order feature, first-order kurtosis, which differs from the IBSI definition. A description of the features is provided in Supplementary Materials. The PyRadiomics documentation [44] provides a complete overview of all radiomics features.

### 2.4. Feature Selection

Unsupervised and supervised feature selection was performed on the training dataset to reduce data dimensionality and the chance of overfitting on the training data. Highly correlated features were assumed to contain overlapping information about the outcome and are therefore considered redundant; thus, for each correlating feature pair, one was selected, and the other was removed. Through absolute pairwise Spearman rank correlation, highly correlating features (>0.85) were determined. The feature with the highest mean absolute correlation with the rest of the dataset was then excluded. Univariate feature selection was performed by fitting a univariate cox model for each individual feature. Afterwards, we selected features based on the individual feature’s association with survival. This was performed by choosing features with a testing association *p*-value (Wald test) lower than the threshold of 0.05 [45]. A false discovery rate (FDR) adjustment was performed on the *p*-values to correct for multiple testing [46]. A 100-repeat 10-fold cross-validation was performed to determine the most prognostic features on average.

### 2.5. Radiomics Model

A multivariable Cox model was trained on the training dataset using the selected features. Afterwards, the model’s prognostic performance was assessed through external validation on the validation dataset. This was performed according to the principles and methods described by Royston and Altman (2013) [47], described in Supplementary Materials. Model discrimination performance was determined through CI. A CI of 0.5 means the predictions are achieved completely randomly, while values near 1 indicate almost perfect discriminative performance. Risk-stratified KM curves were generated for each model, which allowed for visual comparison between models and provided the opportunity to determine how well the cohort could be stratified into risk groups. Three risk groups were determined using threshold values at the 33rd and 66th percentile of the calculated prognostic index (PI). Two log-rank tests were performed to determine the significance of the split of the low- vs. the medium-risk groups, and the medium- vs. the high-risk groups. Predicted survival curves for each risk group were determined. The individual survival curves were estimated using the PI of each patient, which were then averaged over the entire risk group. The observed survival curves and predicted survival curves aligning indicates that the model fits correctly to the data.

### 2.6. Staging, Volume, and Clinical Models

Risk stratification based on TNM8, primary tumour volume, and a model developed from clinical and biological features were compared to the radiomics model’s results. The radiomics feature ‘original_shape_VoxelVolume’ was used as a surrogate for tumour volume. This feature was added to the list of selected features and used to create a separate model [48]. The clinical and biological model was built from a list of known predictors of survival in HNSCC, which can be found in Supplementary Materials. All features had less than 10% of values missing. For any missing values imputation was performed using the ‘missForest’ package in R [49]. This imputation method trains a random forest (RF) model on the existing data to predict the missing values. Imputation was performed separately for the training and validation datasets. Feature selection on the clinical and biological covariates was performed through univariate Cox modelling, selecting univariate significant covariates through chi-square test *p*-values after correcting for multiple testing (FDR) [46]. The significant features were added to the list of radiomics features and used to create separate models. In addition, a combined model using radiomics, tumour volume, and clinical/biological variables was created and validated.

### 2.7. Validation of Existing Radiomics Signatures

Aerts et al. reported on a radiomics signature to predict survival in lung cancer patients which they validated on HNSCC cohorts [50]. This signature was evaluated both on our validation and the full cohort (training and validation), and its performance was compared to the newly proposed signature created. After the necessary preprocessing steps, the four features used by Aerts to establish the signature were extracted from the primary tumour volume. The feature values were multiplied with the β coefficients reported in the article to calculate the linear predictor. To stratify the patients into low- and high-risk groups, the authors used a single cut-off value based on the linear predictor’s median. We applied these cut-offs in order to determine two risk groups and compared these to risk stratification using the median of the linear predictor estimated by our novel models.

### 2.8. Radiomics Quality Score and TRIPOD

For quality assurance, the radiomics quality score (RQS) [51,52] was calculated and transparent reporting of a multivariable prediction model for individual prognosis or diagnosis (TRIPOD) [53] recommendations were followed. A description of these statements and the results can be found in Supplementary Materials Tables S5 and S6.

## 3. Results

### 3.1. Clinical, Biological, and Imaging Characteristics

In total, 666 retrospective and 143 prospective patients were collected and analysed in this study. Table 1 provides an overview of the patient characteristics for both cohorts.

The median follow-up of patients in the training and validation cohort was 63 (49–79 95% CI) and 32 (26–37 95% CI) months, respectively. Two-year survival in the training and validation cohort was 78% and 75%, respectively. A log-rank test between survival curves shows that the difference between cohorts is not significant (*p* = 0.29). KM plots of the cohorts are shown in Supplementary Materials Figure S1. As oropharyngeal carcinoma constituted a significant portion of the dataset (43%/*n* = 294 for training, 36% *n* = 51 for validation), we decided to build separate models for this group of patients (including both p16+ and p16−). A description of this model, along with the results, can be found in Supplementary Materials. Supplementary Materials Figure S2 shows an overview of the different parameters used for image acquisition and reconstruction in the training and validation datasets.

### 3.2. Model Results

We extracted 1198 radiomics features from the primary tumour volume on all CT images. After unsupervised feature selection, 204 features remained. In total, 11 features were selected by supervised selection as being the most predictive of OS in the training cohort. The first two features were kurtosis, a first-order statistics feature, and sphericity, a shape feature. The next four features are all LoG-filtered texture features consisting of GLSZM gray level non-uniformity, GLDM entropy, GLRLM run entropy, and GLDM low gray-level emphasis. Finally, five wavelet-filtered texture features were included: four differently wavelet-filtered GLSZM zone entropy features and GLRLM low gray-level-run emphasis. All selected features were IBSI compliant, except for the first-order statistics feature. Supplementary Materials Table S3 shows an overview of the feature names. The slope of the PI in validation was 1.35, and a log-rank test indicated this slope was not significantly different from a slope of 1 (*p*-value of 0.38). This indicates the model calibrates well, meaning the predicted and the expected outcome proportions for a certain testing population match. The joint test of all predictors with the offset of the PI gives a *p*-value of 0.86, indicating there is no evidence of a lack of fit on the validation cohort.

Supplementary Materials Figure S3 depicts KM survival curves for the combined training and validation cohort after stratification in two risk groups (*p* < 0.01) using the Aerts et al. (2014) signature [50], with a CI of 0.66. For some patients, one or more of the required features failed to extract due to the small size of the volume. Therefore, the calculation of the signature was not possible in all available patients, resulting in 633 patients in the training cohort and 139 patients in the validation cohort. The performance of the signature in this study is similar to the reported validation performance on the lung dataset (CI of 0.65) but slightly lower than the performance on the two H&N datasets (both CI of 0.69).

**Figure 2:**
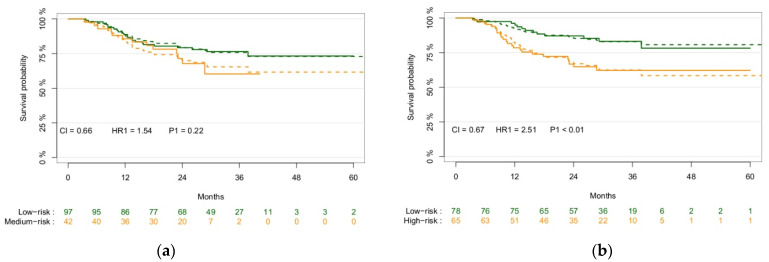
Kaplan–Meier survival plots of the validation cohort (*n* = 139) stratified based on the previously created signature (**a**) and the newly created signature (**b**), showing the *p*-value of the split between risk groups and model performance through the CI and the HR between the risk groups. The solid lines represent the observed survival curves, and the dashed line the corresponding predicted survival curves.

Figure 2 shows KM survival graphs of the validation cohort split using the previously created signature [50] and the radiomics-only signature from this study. While the CI values of the model performances are similar (0.66 and 0.67, respectively), the split and hazard ratios are significantly better using the newly created signature (*p* = 0.22 vs. *p* < 0.01).

**Figure 3:**
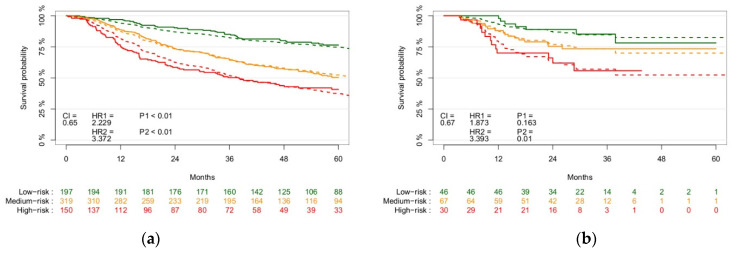
Kaplan–Meier survival curves of the training (**a**), *n* = 666) validation ((**b**), *n* = 143) patient cohorts stratified into low-, medium-, and high-risk groups, showing log-rank test *p*-value of the split between risk groups and the CI of the radiomics features-based model performance. The solid lines represent the observed survival curves, and the dashed line the corresponding predicted survival curves.

Figure 3 shows the KM survival graphs of the training and validation cohorts with a CI of 0.65 and 0.67 in training and validation, respectively. The *p*-values of the log-rank test of the low and medium, and medium and high split were <0.01 for both in training, and 0.163 and 0.01 in validation, respectively. This CI is similar to stratification based on tumour volume alone (CDI of 0.68), shown in Supplementary Materials Figure S4. However, the shape of the KM curve shows tumour volume is very poor in discerning three distinct risk groups. It is significantly lower than stratification based on TNM8 (CI of 0.74), shown in Supplementary Materials Figure S5.

**Figure 4:**
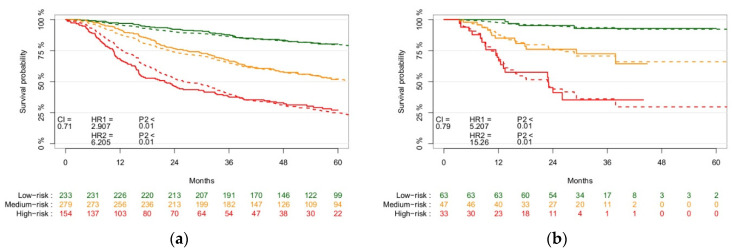
Kaplan–Meier survival cohorts of the training ((**a**), *n* = 666) validation ((**b**), *n* = 143) patient cohorts stratified into low-, medium-, and high-risk groups based on radiomics, tumour volume, clinical, and biological parameters, showing the *p*-value of the split between risk groups and CI of the model performance. The solid lines represent the observed survival curves, and the dashed line the corresponding predicted survival curves.

An overview of the clinical and biological features selected is shown in Table 2. The clinical features selected through univariate feature selection were TNM8 (higher stage has worse prognosis), age at diagnosis (higher age has worse prognosis), ACE-27 comorbidity score (higher score has worse prognosis), smoking pack-years (higher pack-years has worse prognosis), and alcohol consumption at the time of diagnosis (current has worst prognosis), and the biological features were p16-status (p16-negative has worse prognosis) and clinical Hb level at baseline (lower Hb level has worse prognosis). Supplementary Materials Figure S6 shows the KM curve stratified based on these clinical and biological features, with a CI of 0.73. Figure 4 shows KM survival curves of the validation cohort after stratification based on tumour volume, the selected clinical and biological parameters, and the selected radiomics features, with a CI of 0.71 and 0.79 in training and validation, respectively. The *p*-value of the log-rank test of the low and medium, and medium and high split were both <0.01 in both training and validation.

For the oropharynx patient cohort, eight features were selected as being the most predictive of OS, consisting of one first-order statistics feature, two shape features, three wavelet-filtered texture features, and two LoG-filtered texture features. All selected features were IBSI compliant. Supplementary Materials Table S4 shows an overview of the features. The slope of the PI in the validation was 3.01. A log-rank test indicates with certainty the slope in the validation is larger than unity (*p*-value of 0.04). The *p*-value for the joint test of all predictors with the PI offset is 0.12. This indicates that there is no proof of a lack of fit on the validation cohort. Kaplan–Meier survival curves of the prospective oropharynx cohort split based on radiomics features are shown in Supplementary Materials Figure S7.

**Figure 5:**
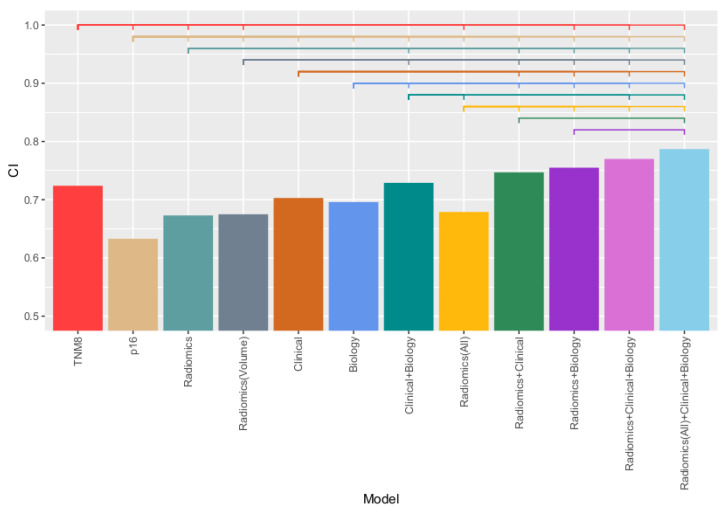
Bar plot of the various models validated on the validation cohort (*n* = 143). The *y*-axis indicates CI value, while the coloured bars above the bar show significant differences between models, with an indent meaning the model is significantly different, and no indent meaning no significant difference was found.

A full overview of the different combinations of models, with discrimination performance and hazard ratios for each model, is provided in Table 3. In addition, Figure 5 provides an overview of the CI indices of the validation results.

From Table 3 and Figure 5, it can be observed that in the prospective cohort radiomics alone did not perform better than TNM8 (CI of 0.67 and 0.74, respectively, *p* < 0.01). Combining TNM8 and radiomics resulted in higher performance than both separately, with a CI of 0.77. In combination with both clinical parameters and tumour volume, the highest discrimination performance was found (CI of 0.79). Similarly, oropharynx radiomics did not perform better than TNM8 (CI of 0.82 vs. 0.86, *p* < 0.01), but when combining both radiomics and TNM8, the highest performance in the validation cohort was achieved (CI of 0.90).

## 4. Discussion

For advanced tumours such as those investigated in this study, being able to discern groups of poor versus good performing patients is key for personalised decision making. In this international, multicentre study, we created a multifactorial prediction model, including radiomics features derived from the primary tumour volume that can significantly stratify advanced HNSCC patients in good, average, and poor prognostic groups, with a CI of 0.79 as validation on a prospective cohort. These groups could be used in clinical decision making and for selecting patients for (de-)escalation trials and/or adjuvant treatment. While radiomics alone was not able to improve on TNM8, adding radiomics features to a model including TNM8, clinical, and biological variables improved the prognostic performance, significantly increasing CI from 0.73 to 0.79. We can therefore recommend adding these variables to the current clinical implementation of TNM8. 

These results coincide with other works reporting on the complementary value of radiomics in predictive modelling for HNSCC [10,54]. The performance of the model based solely on radiomics, with a CI of 0.67, matches those of similar studies which investigate OS [10,11,12,13,14]. However, compared to these studies, this study investigates over 800 patients from multiple centres, whose data were partially collected prospectively. The largest discrepancy is with the study by Cozzi et al. (2019), which found a high CI of 0.90 in validation [12]. While their methodology is sound, as the writers explain themselves the number of patients (*n* = 110) from a single centre does make these results less significant. Haider et al. (2020) had the largest cohort of 306 patients with a CI of 0.58 in validation [14]. This result was found on an external cohort, and while lower, it is more in line with the result we found in external validation.

In total, 11 radiomics features were selected as being univariately most predictive of OS. The first two selected features were kurtosis, a first-order statistics feature that measures the ‘peakness’ of the distribution of pixel intensity values, and sphericity, a shape feature that measures the likeness of the ROI to a sphere. Sphericity being selected implies fewer spherical tumours may have a worse prognosis. The next four features are all LoG-filtered texture features consisting of GLSZM gray level non-uniformity, a feature which measures the variability of gray-level intensity values, GLDM entropy, and GLRLM run entropy, which both measure the heterogeneity in texture patterns, and GLDM low gray-level emphasis, which measures the concentration of low-intensity values. Finally, five wavelet-filtered texture features were included: four differently wavelet-filtered GLSZM zone entropy features, which measure the heterogeneity in texture patterns, and GLRLM low gray-level-run emphasis, which measures the concentration of low-intensity values. Most of these features are linked to heterogeneity, reinforcing the theory that tumour heterogeneity correlates with a worse prognosis [55,56].

For most tested models, we found a higher validation accuracy than training accuracy. This may be caused by the relatively smaller size of the validation dataset, which means the result is more prone to variance, which is reflected in the larger confidence intervals, especially for the smaller oropharyngeal analysis. Another contributing factor could be that the training dataset contains relatively more ‘hard’ cases than the validation dataset. In this paper, we chose to validate on a prospectively collected dataset, which is for data splitting purposes an arbitrary reason. In a more balanced dataset with more similar patient datasets, the discrepancy between training and validation may be lower.

Instead of using radiotherapy planning images only, which is conventional for radiomics studies, this study used diagnostic CT images as well, which are made routinely for any patient showing a locally advanced HNSCC. From these images radiomics features can be extracted in a semi-automatic fashion, making clinical application easy. In addition, the combined model was made using simple variables that are routinely determined in a clinical setting for every patient (TNM8 stage, ACE-27 comorbidity status, smoking, and alcohol habits). As a result, it would be relatively easy to implement the presented models in a clinical environment. For the next step, the created model could be tested in a clinical trial. However, as differences in scanners, scan settings, and acquisition settings have proven to significantly affect feature reproducibility, a prospective study where these variables are controlled may be required to further validate model performance.

Radiomics performs an estimation of the tumour volume using a 3D segmentation, as opposed to conventional methods of measuring tumour volume to predict survival. This single feature was found to be significantly predictive of OS, albeit with lower performance, compared to TNM8 or the model based on radiomics features but was not chosen in the multivariable model. The main reason for this is the interaction with other features in the correlation dimensionality reduction step. Volume has a high correlation with other features, mostly shape features, and is therefore removed from the feature dataset before univariate selection is performed, revealing a shortcoming of this feature reduction step. However, the information provided by this feature should be retained in the remaining uncorrelated features.

The radiomics model in this study shows better performance in stratifying patients in risk groups in the validation dataset when compared to the previously created and validated signature [50]. One large discrepancy between these models is the risk stratification: the previously developed signature was created with two risk groups instead of three. Most importantly, it was built on lung cancer. The difference in performance on different tumour sites demonstrates that prognostic models should be developed on specific tumour sites and stages, and with relevant clinical risk groups in mind.

While the amount of data used in this study was higher than most published radiomics studies, this was partially achieved by pooling data from different HNSCC sites. Separating these regions resulted in very small datasets in either or both training and validation sets. While we had sufficient data to train an oropharynx model and found a relatively high performance of the model using radiomics features of 0.82 CI in validation, the number of patients, and particularly the number of events, of the validation dataset was relatively limited. Collecting more data from an individual tumour site would most likely result in more representative models. In addition, the patients in this study received different treatments. This significantly affects survival chance and is therefore a major limitation. Similar to tumour region, separate models according to treatment would be preferred. However, treatment is heavily linked to the region of the tumour, as, for example, the majority of surgeries were performed for oral cavity patients.

Compared to extracting radiomics features from just the primary tumour volume, TNM8 staging takes information from the primary tumour (T-stage), involvement in lymph nodes (N-stage), and the extent of metastisation (M-stage) into consideration. In addition, depending on the tumour region, additional information such as p16-status as a surrogate for HPV involvement, depth of invasion in surrounding tissues, and presence of extranodal extension are important. The addition of radiomics features derived from lymph node metastases can potentially improve the results. This would require a multifactorial model with a binary condition for the lymph node stage and would only incorporate features of those patients who have lymph node metastases.

Imaging artefacts caused by dental implants may have affected the performance of the radiomics model. The artefacts make segmentation difficult but also affect the radiomics features extracted from these images. While there was a limit on the number of artefacts allowed on images during patient selection, methods to reduce the artefacts may be considered for future studies. In addition, variability caused by the manual segmentation of tumours by different experts at each institute may have also affected model performance. Previous research has shown that inter- and intraobserver variability can possibly cause large differences in delineated volumes [57]. For shape and size radiomics features, this can cause a large decrease in their use and may affect other features to a lesser degree. The repeatability of deep-learning-based automatic segmentation methods will be able to negate interobserver variabilities in the future [58].

To compensate for interobserver variability in the current project, each centre performed delineations either directly by, or under the supervision of, expert radiologists or radiation oncologists. Additionally, although delineations were performed according to local protocols, European guidelines are largely aligned, limiting the interobserver effects on the delineated structures. Conversely, in a clinical application of the proposed model at different institutes, interobserver variabilities will be an inevitability. The discriminative performance the model has shown despite these issues strengthens the potential of application in a clinical setting.

## 5. Conclusions

A multifactorial prognostic model for stage III-IVB HNSCC (TNM7th edition) based on simple variables available for every patient and including CT radiomics features is able to very accurately predict OS and to significantly discern different risk groups. The multifactorial model was found to have higher predictive performance than the current gold standard of TNM8. This could be useful in treatment (de-)escalation trials and clinical decision support.

## Figures and Tables

**Table 1:** Patient characteristics overview for retrospective and prospective patient cohorts. HN = head and neck, RT = radiotherapy, CH = chemotherapy, CRT = chemoradiotherapy, ECOG PS = eastern cooperative oncology group performance status.

Study			Retrospective (*n* = 666)	Prospective (*n* = 143)	*p*-Value
Sex (% male/*n*)			72/482	65/93	*p* = 0.10
Age (Median/range)			63/	64/	*p* = 0.17
			29–89	38–93	
HN tumour site (%/*n*)		-Hypopharynx	15/96	15/21	*p* = 0.93
		-Oropharynx	43/289	36/51	*p* = 0.11
		-Oral cavity	15/100	29/42	*p* < 0.01
		-Larynx	27/181	20/29	*p* = 0.11
p16+ Oropharynx (%/*n*)			22/146	26/37	*p* = 0.36
Stage TNM7th edition (%/*n*)		-III	31/206	28/40	*p* = 0.55
		-IVa	59/390	67/96	*p* = 0.07
		-IVb	10/70	5/7	*p* = 0.06
Stage TNM8th edition(%/*n*)	p16+ oropharynx	-I	11/74	12/17	*p* = 0.90
		-II	6/42	9/13	*p* = 0.31
		-III	5/30	5/7	*p* = 1
	Non-oropharynx/p16-oropharynx	-III	25/169	28/40	*p* = 0.59
		-IVa	37/248	38/54	*p* = 0.98
		-IVb	16/103	8/12	*p* = 0.04
Treatment (% of patients received type of treatment/*n*)		-RT only	29/191	15/22	*p* < 0.01
		-Surgery only	5/34	4/5	*p* < 0.01
		-CRT	37/245	36/51	*p* = 0.55
		-Surgery + RT	15/102	24/34	*p* = 0.16
		-Surgery + CH + RT	14/93	12/17	*p* = 0.60
Order of CH (% of CH patients/*n*)		-Adjuvant	15/51	12/8	*p* = 0.61
		-Concomitant	81/273	84/57	*p* = 0.64
		-Induction	4/15	4/3	*p* = 1
ACE-27 Comorbidity (%/*n*)		=0	30/204	38/52	*p* = 0.20
		=1	41/272	38/52	*p* = 0.37
		=2	20/133	16/21	*p* = 0.18
		=3	9/57	8/11	*p* = 0.86
Smoking (%/*n*)		-Current	52/350	40/55	*p* = 0.01
		-Former	36/237	33/45	*p* = 0.44
		-Never	12/79	27/37	*p* < 0.01
Pack years (Median/range)			35/0–174	30/0–220	*p* = 1
Alcohol consumption (%/*n*)		-Current	66/445	48/67	*p* < 0.01
		-Former	13/84	12/17	*p* = 1
		-Never	21/137	40/55	*p* < 0.01
ECOG PS (%/*n*)		=0	39/262	49/68	*p* < 0.01
		=1	16/106	43/59	*p* < 0.01
		=2	3/21	8/11	*p* = 0.22
		=3	1/4	-	*p* = -
		=NA	41/273	4/5	*p* < 0.01
Hb level (Median/range)			8.8/5.0–15.1	8.7/4.8-14.0	*p* = 0.27

**Table 2:** Selected clinical and biological features in the clinical, biological, and combined models, with univariate model coefficient, hazard ratio, and significance to outcome shown.

Feature Name	Model Coefficient	Hazard Ratio	*p*-Value
TNM8	0.76	2.14	<0.01
Age	0.034	1.035	<0.01
ACE-27 comorbidity score	0.28	1.33	<0.01
Pack years	0.005	1.005	0.02
Alcohol at diagnosis	0.47	1.61	<0.01
P16-status	−1.3	0.27	<0.01
Haemoglobin level	−0.3	0.74	<0.01

**Table 3:** Performance overview of all trained and/or validated models, showing Harrell’s CI and HR values for each model. The left side shows the models for the full patient cohort, both training (*n* = 666) and validation (*n* = 143), the right the oropharynx patient cohort, both training (*n* = 294) and validation (*n* = 51). * indicates no HR could be calculated, as the low-risk group did not have any events recorded.

	Full Patient Cohort						Oropharynx Patient Cohort					
	Training			Validation			Training			Validation		
Model	CI (95% CI)	HR 1 vs. 2 (95% CI)	HR 1 vs. 3 (95% CI)	CI (95% CI)	HR 1 vs. 2 (95% CI)	HR 1 vs. 3 (95% CI)	CI (95% CI)	HR 1 vs. 2 (95% CI)	HR 1 vs. 3 (95% CI)	CI (95% CI)	HR 1 vs. 2 (95% CI)	HR 1 vs. 3 (95% CI)
Staging TNM8	0.65 (0.64–0.65)	1.82 (1.40–2.35)	3.12 (2.32–4.21)	0.74 (0.73–0.75)	5.01 (2.11–11.85)	14.03 (5.16–38.17)	0.71 (0.69–0.72)	2.50 (1.62–3.87)	5.16 (3.24–8.23)	0.86 (0.81–0.87)	9.12 (1.28–64.90)	30.15 (4.97–182.90)
Radiomics	0.65 (0.64–0.65)	2.22 (1.64–3.03)	3.37 (2.41–4.72)	0.67 (0.66–0.69)	1.87 (0.78–4.52)	3.39 (1.33–8.64)	0.68 (0.67–0.69)	2.36 (1.45–3.86)	3.80 (2.21–6.52)	0.82 (0.78–0.85)	-*	-*
Radiomics + Staging	0.68 (0.68–0.69)	2.49 (1.77–3.44)	4.60 (3.24–6.53)	0.77 (0.75–0.78)	8.54 (1.97–37.98)	29.35 (6.73–127.94)	0.73 (0.73–0.74)	3.97 (2.20–7.18)	7.87 (4.39.27)	0.90 (0.88–0.92)	-*	-*
Radiomics (Volume)	0.62 (0.62–0.62)	1.48 (1.08–2.03)	3.17 (2.16–4.66)	0.68 (0.66–0.69)	1.23 (0.54–2.78)	7.98 (2.85–22.31)	0.64 (0.63–0.64)	1.81 (1.10–2.99)	3.29 (1.82–5.92)	0.87 (0.84–0.90)	-*	-*
Clinical	0.66 (0.66–0.67)	2.37 (1.76–3.19)	3.25 (2.40–4.40)	0.70 (0.69–0.72)	3.66 (1.40–9.54)	5.37 (2.10–13.72)	0.73 (0.72–0.74)	3.80 (2.18–6.63)	8.27 (4.82–14.18)	0.84 (0.81–0.87)		
Biological	0.63 (0.63–0.63)	2.83 (1.95–4.09)	3.94 (2.71–5.74)	0.70 (0.68–0.71)	13.03 (1.74–97.73)	23.19 (3.08–174.46)	0.68 (0.68–0.69)	4.28 (2.79–6.56)	6.74 (0.91–49.82)	0.84 (0.80–0.89)	*	*
Clinical + Biological	0.67 (0.66–0.67)	2.71 (1.95–3.75)	4.17 (3.00–5.78)	0.73 (0.72–0.74)	8.21 (2.37–28.39)	10.10(2.97–34.36)	0.74 (0.74–0.75)	3.82 (2.16–6.76)	8.66 (5.08–14.76)	0.88 (0.85–0.90)	-*	-*
Radiomics (includes volume)	0.65 (0.65–0.66)	1.78 (1.32–2.42)	3.64 (2.61–5.08)	0.68 (0.67–0.69)	2.19 (0.92–5.26)	3.84 (1.48–9.95)	0.68 (0.67–0.69)	2.47 (1.50–4.06)	3.94 (2.28–6.82)	0.82 (0.78–0.86)	-*	-*
Radiomics + Clinical	0.69 (0.69–0.70)	2.94 (2.15–4.03)	4.79 (3.45–6.67)	0.74 (0.74–0.76)	4.65 (1.86–17.16)	11.38 (3.84–33.74)	0.73 (0.72–0.74)	3.80 (2.18–6.64)	8.27 (4.82–14.18)	0.84 (0.81–0.87)	*	*
Radiomics + Biological	0.68 (0.68–0.68)	2.89 (2.04–4.08)	5.03 (3.52–7.17)	0.76 (0.74–0.77)	6.49 (1.91–22.06)	13.74 (3.96–47.66)	0.74 (0.74–0.75)	3.61 (2.13–6.12)	6.85 (4.12–11.39)	0.91 (0.90–0.93)	*	*
Radiomics + Clinical + Biological	0.70 (0.70–0.70)	3.04 (2.17–4.27)	5.82 (4.10–8.28)	0.77 (0.77–0.78)	8.17 (2.36–28.24)	13.17 (3.86–44.85)	0.77 (0.77–0.78)	4.77 (2.65–8.60)	12.53 (7.03–22.31)	0.88 (0.85–0.90)	-*	-*
Radiomics (includes volume) + Clinical + Biological	0.71 (0.71–0.71)	2.91 (2.11–4.01)	6.21 (4.44–8.68)	0.79 (0.78–0.80)	5.21 (1.70–15.98)	15.26 (5.14–45.32)	0.77 (0.76–0.77)	6.11 (3.23–11.53)	15.40 (8.17–29.03)	0.87 (0.84–0.89)	-*	-*
p16-status	-	-	-	-	-	-	0.67 (0.67–0.68)	4.3 (2.81–6.59)	-	0.82 (0.78–0.85)	19.8 (2.38–165)	-
Aerts. 2014 [50]	0.61 (0.61–0.61)	1.65 (1.30–2.09)	-	0.66	1.54 (0.77–3.06)	-	0.65 (0.64–0.66)	1.90 (1.3–2.77)	-	0.68 (0.63–0.73)	-*	-

## Data Availability

The data presented in this study are available on request from the corresponding author. The data are not publicly available due to an agreement within the BD2Decide consortium that the data will be closed access for a defined period to allow the participants of the consortium to utilise the data for publication purposes first.

## Supplementary Materials

The following are available online at https://www.mdpi.com/article/10.3390/cancers13133271/s1, Figure S1: Retrospective vs. prospective survival curves, Table S1: Patient treatment characteristics, Figure S2: Imaging parameter graphs, Figure S3: Signature validation on combined dataset, Figure S4: Tumour volume stratified KM curves, Figure S5: TNM8 stratified KM curves, Figure S6: Clinical and biological features stratified KM curves, Figure S7: Radiomics model stratified KM curves of the oropharynx sub-cohort, Table S2: Table of used R packages, Table S3: Selected radiomics features, Table S4: Selected radiomics features for the oropharynx sub-cohort, Table S5: RQS checklist, Table S6: TRIPOD statement checklist.

## References

[1] Bray F., Ferlay J., Soerjomataram I., Siegel R.L., Torre L.A., Jemal A. (2018). Global cancer statistics 2018: GLOBOCAN estimates of incidence and mortality worldwide for 36 cancers in 185 countries. CA Cancer J. Clin..

[2] Mehra R., Ang K.K., Burtness B. (2012). Management of human papillomavirus-positive and human papillomavirus-negative head and neck cancer. Semin. Radiat. Oncol..

[3] Lubin J.H., Purdue M., Kelsey K., Zhang Z.F., Winn D., Wei Q., Talamini R., Dabrowska N.S., Sturgis E.M., Smith E. (2009). Total exposure and exposure rate effects for alcohol and smoking and risk of head and neck cancer: A pooled analysis of case-control studies. Am. J. Epidemiol..

[4] Lydiatt W., O’Sullivan B., Patel S. (2018). Major Changes in Head and Neck Staging for 2018. Am. Soc. Clin. Oncol. Educ. Book.

[5] Lydiatt W.M., Patel S.G., O’Sullivan B., Brandwein M.S., Ridge J.A., Migliacci J.C., Loomis A.M., Shah J.P. (2017). Head and Neck cancers-major changes in the American Joint Committee on cancer eighth edition cancer staging manual. CA Cancer J. Clin..

[6] Qi Z., Barrett T., Parikh A.S., Tirosh I., Puram S.V. (2019). Single-cell sequencing and its applications in head and neck cancer. Oral Oncol..

[7] Mroz E.A., Rocco J.W. (2016). Intra-tumor heterogeneity in head and neck cancer and its clinical implications. World J. Otorhinolaryngol. Head Neck Surg..

[8] Bogowicz M., Riesterer O., Ikenberg K., Stieb S., Moch H., Studer G., Guckenberger M., Lang S.T. (2017). Computed Tomography Radiomics Predicts HPV Status and Local Tumor Control After Definitive Radiochemotherapy in Head and Neck Squamous Cell Carcinoma. Int. J. Radiat. Oncol. Biol. Phys..

[9] Mukherjee P., Cintra M., Huang C., Zhou M., Zhu S., Colevas A.D., Fischbein N., Gevaert O. (2020). CT-based Radiomic Signatures for Predicting Histopathologic Features in Head and Neck Squamous Cell Carcinoma. Radiol. Imaging Cancer.

[10] Ou D., Blanchard P., Rosellini S., Levy A., Nguyen F., Leijenaar R.T.H., Garberis I., Gorphe P., Bidault F., Ferté C. (2017). Predictive and prognostic value of CT based radiomics signature in locally advanced head and neck cancers patients treated with concurrent chemoradiotherapy or bioradiotherapy and its added value to Human Papillomavirus status. Oral Oncol..

[11] Xie C., Yang P., Zhang X., Xu L., Wang X., Li X., Zhang L., Xie R., Yang L., Jing Z. (2019). Sub-region based radiomics analysis for survival prediction in oesophageal tumours treated by definitive concurrent chemoradiotherapy. EBioMedicine.

[12] Cozzi L., Franzese C., Fogliata A., Franceschini D., Navarria P., Tomatis S., Scorsetti M. (2019). Predicting survival and local control after radiochemotherapy in locally advanced head and neck cancer by means of computed tomography based radiomics. Strahlenther. Onkol..

[13] Liu Z., Cao Y., Diao W., Cheng Y., Jia Z., Peng X. (2020). Radiomics-based prediction of survival in patients with head and neck squamous cell carcinoma based on pre- and post-treatment (18)F-PET/CT. Aging.

[14] Haider S.P., Zeevi T., Baumeister P., Reichel C., Sharaf K., Forghani R., Kann B.H., Judson B.L., Prasad M.L., Burtness B. (2020). Potential Added Value of PET/CT Radiomics for Survival Prognostication beyond AJCC 8th Edition Staging in Oropharyngeal Squamous Cell Carcinoma. Cancers.

[15] Head, MD Anderson Cancer Center, Neck Quantitative Imaging Working Group (2018). Investigation of radiomic signatures for local recurrence using primary tumor texture analysis in oropharyngeal head and neck cancer patients. Sci. Rep..

[16] Li W., Wei D., Wushouer A., Cao S., Zhao T., Yu D., Lei D. (2020). Discovery and Validation of a CT-Based Radiomic Signature for Preoperative Prediction of Early Recurrence in Hypopharyngeal Carcinoma. Biomed. Res. Int..

[17] Bogowicz M., Tanadini-Lang S., Guckenberger M., Riesterer O. (2019). Combined CT radiomics of primary tumor and metastatic lymph nodes improves prediction of loco-regional control in head and neck cancer. Sci. Rep..

[18] Zhai T.T., Langendijk J.A., van Dijk L.V., Halmos G.B., Witjes M.J.H., Oosting S.F., Noordzij W., Sijtsema N.M., Steenbakkers R.J.H.M. (2019). The prognostic value of CT-based image-biomarkers for head and neck cancer patients treated with definitive (chemo-)radiation. Oral Oncol..

[19] Leger S., Zwanenburg A., Leger K., Lohaus F., Linge A., Schreiber A., Kalinauskaite G., Tinhofer I., Guberina N., Guberina M. (2020). Comprehensive Analysis of Tumour Sub-Volumes for Radiomic Risk Modelling in Locally Advanced HNSCC. Cancers.

[20] Agarwal J.P., Sinha S., Goda J.S., Joshi K., Mhatre R., Kannan S., Laskar S.G., Gupta T., Murthy V., Budrukkar A. (2020). Tumor radiomic features complement clinico-radiological factors in predicting long-term local control and laryngectomy free survival in locally advanced laryngo-pharyngeal cancers. Br. J. Radiol..

[21] Tang S., Ou J., Liu J., Wu Y.P., Wu C.Q., Chen T.W., Zhang X.M., Li R., Tang M.J., Yang L.Q. (2021). Application of contrast-enhanced CT radiomics in prediction of early recurrence of locally advanced oesophageal squamous cell carcinoma after trimodal therapy. Cancer Imaging.

[22] Wu W., Ye J., Wang Q., Luo J., Xu S. (2019). CT-Based Radiomics Signature for the Preoperative Discrimination Between Head and Neck Squamous Cell Carcinoma Grades. Front. Oncol..

[23] Zhang M.H., Cao D., Ginat D.T. (2021). Radiomic Model Predicts Lymph Node Response to Induction Chemotherapy in Locally Advanced Head and Neck Cancer. Diagnostics.

[24] Zhai T.T., Wesseling F., Langendijk J.A., Shi Z., Kalendralis P., van Dijk L.V., Hoebers F., Steenbakkers R.J.H.M., Dekker A., Wee L. (2021). External validation of nodal failure prediction models including radiomics in head and neck cancer. Oral Oncol..

[25] Guo R., Guo J., Zhang L., Qu X., Dai S., Peng R., Chong V.F.H., Xian J. (2020). CT-based radiomics features in the prediction of thyroid cartilage invasion from laryngeal and hypopharyngeal squamous cell carcinoma. Cancer Imaging.

[26] Liu Y., Shi H., Huang S., Chen X., Zhou H., Chang H., Xia Y., Wang G., Yang X. (2019). Early prediction of acute xerostomia during radiation therapy for nasopharyngeal cancer based on delta radiomics from CT images. Quant. Imaging Med. Surg..

[27] van Dijk L.V., Langendijk J.A., Zhai T.T., Vedelaar T.A., Noordzij W., Steenbakkers R.J.H.M., Sijtsema N.M. (2019). Delta-radiomics features during radiotherapy improve the prediction of late xerostomia. Sci. Rep..

[28] Sheikh K., Lee S.H., Cheng Z., Lakshminarayanan P., Peng L., Han P., McNutt T.R., Quon H., Lee J. (2019). Predicting acute radiation induced xerostomia in head and neck Cancer using MR and CT Radiomics of parotid and submandibular glands. Radiat. Oncol..

[29] Ou J., Li R., Zeng R., Wu C.Q., Chen Y., Chen T.W., Zhang X.M., Wu L., Jiang Y., Yang J.Q. (2019). CT radiomic features for predicting resectability of oesophageal squamous cell carcinoma as given by feature analysis: A case control study. Cancer Imaging.

[30] Huang C., Cintra M., Brennan K., Zhou M., Colevas A.D., Fischbein N., Zhu S., Gevaert O. (2019). Development and validation of radiomic signatures of head and neck squamous cell carcinoma molecular features and subtypes. EBioMedicine.

[31] Zhu Y., Mohamed A.S.R., Lai S.Y., Yang S., Kanwar A., Wei L., Kamal M., Sengupta S., Elhalawani H., Skinner H. (2019). Imaging-Genomic Study of Head and Neck Squamous Cell Carcinoma: Associations Between Radiomic Phenotypes and Genomic Mechanisms via Integration of The Cancer Genome Atlas and The Cancer Imaging Archive. JCO Clin. Cancer Inform..

[32] Cavalieri S., De Cecco L., Brakenhoff R.H., Serafini M.S., Canevari S., Rossi S., Lanfranco D., Hoebers F.J.P., Wesseling F.W.R., Keek S. (2020). Development of a multiomics database for personalized prognostic forecasting in head and neck cancer: The Big Data to Decide EU Project. Head Neck.

[33] Perez L.L., Hernández L., Ottaviano M., Martinelli E., Poli T., Licitra L., Arredondo M.T., Fico G. BD2Decide: Big Data and Models for Personalized Head and Neck Cancer Decision Support. Proceedings of the 2019 IEEE 32nd International Symposium on Computer-Based Medical Systems (CBMS).

[34] Ramroth H., Schoeps A., Rudolph E., Dyckhoff G., Plinkert P., Lippert B., Feist K., Delank K.W., Scheuermann K., Baier G. (2011). Factors predicting survival after diagnosis of laryngeal cancer. Oral Oncol..

[35] Faye-Lund H., Abdelnoor M. (1996). Prognostic factors of survival in a cohort of head and neck cancer patients in Oslo. Eur. J. Cancer B Oral Oncol..

[36] Smith E.M., Rubenstein L.M., Haugen T.H., Pawlita M., Turek L.P. (2012). Complex etiology underlies risk and survival in head and neck cancer human papillomavirus, tobacco, and alcohol: A case for multifactor disease. J. Oncol..

[37] Shuster J.J. (1991). Median follow-up in clinical trials. J. Clin. Oncol..

[38] Breen S.L., Publicover J., de Silva S., Pond G., Brock K., O’Sullivan B., Cummings B., Dawson L., Keller A., Kim J. (2007). Intraobserver and interobserver variability in GTV delineation on FDG-PET-CT images of head and neck cancers. Int. J. Radiat. Oncol. Biol. Phys..

[39] Steenbakkers R.J.H.M., Duppen J.C., Fitton I., Deurloo K.E.I., Zijp L.J., Comans E.F.I., Uitterhoeve A.L.J., Rodrigus P.T.R., Kramer G.W.P., Bussink J. (2006). Reduction of observer variation using matched CT-PET for lung cancer delineation: A three-dimensional analysis. Int. J. Radiat. Oncol. Biol. Phys..

[40] Rasch C.R.N., Steenbakkers R.J.H.M., Fitton I., Duppen J.C., Nowak P.J.C.M., Pameijer F.A., Eisbruch A., Kaanders J.H.A.M., Paulsen F., van Herk M. (2010). Decreased 3D observer variation with matched CT-MRI, for target delineation in Nasopharynx cancer. Radiat. Oncol..

[41] van Griethuysen J.J.M., Fedorov A., Parmar C., Hosny A., Aucoin N., Narayan V., Tan R.G.H.B., Robin J.C.F., Pieper S., Aerts H.J.W.L. (2017). Computational Radiomics System to Decode the Radiographic Phenotype. Cancer Res..

[42] Hatt M., Vallieres M., Visvikis D., Zwanenburg A. (2018). IBSI: An international community radiomics standardization initiative. J. Nucl. Med..

[43] Zwanenburg A., Vallières M., Abdalah M.A., Aerts H.J.W., Andrearczyk V., Apte A., Ashrafinia S., Bakas S., Beukinga R.J., Boellaard R. (2020). The Image Biomarker Standardization Initiative: Standardized Quantitative Radiomics for High-Throughput Image-based Phenotyping. Radiology.

[44] Community P. https://pyradiomics.readthedocs.io/en/latest/features.html2016.

[45] Emura T., Matsui S., Chen H.Y. (2019). compound.Cox: Univariate feature selection and compound covariate for predicting survival. Comput. Methods Programs Biomed..

[46] Benjamini Y., Hochberg Y. (1995). Controlling the False Discovery Rate: A Practical and Powerful Approach to Multiple Testing. J. Royal Stat. Soc. Ser. B (Methodol.).

[47] Royston P., Altman D.G. (2013). External validation of a Cox prognostic model: Principles and methods. BMC Med. Res. Methodol..

[48] Welch M.L., McIntosh C., Kains B.H., Milosevic M.F., Wee L., Dekker A., Huang S.H., Purdie T.G., O’Sullivan B., Aerts H.J.W.L. (2019). Vulnerabilities of radiomic signature development: The need for safeguards. Radiother. Oncol..

[49] Stekhoven D.J., Bühlmann P. (2011). MissForest—Non-parametric missing value imputation for mixed-type data. Bioinformatics.

[50] Aerts H.J.W.L., Velazquez E.R., Leijenaar R.T.H., Parmar C., Grossmann P., Carvalho S., Bussink J., Monshouwer R., Kains B.H., Rietveld D. (2014). Decoding tumour phenotype by noninvasive imaging using a quantitative radiomics approach. Nat. Commun..

[51] Lambin P., Leijenaar R.T.H., Deist T.M., Peerlings J., de Jong E.E.C., van Timmeren J., Sanduleanu S., Larue R.T.H.M., Even A.J.G., Jochems A. (2017). Radiomics: The bridge between medical imaging and personalized medicine. Nat. Rev. Clin. Oncol..

[52] Sanduleanu S., Woodruff H.C., de Jong E.E.C., van Timmeren J.E., Jochems A., Dubois L., Lambin P. (2018). Tracking tumor biology with radiomics: A systematic review utilizing a radiomics quality score. Radiother. Oncol..

[53] Collins G.S., Reitsma J.B., Altman D.G., Moons K.G. (2015). Transparent Reporting of a multivariable prediction model for Individual Prognosis Or Diagnosis (TRIPOD). Ann. Intern. Med..

[54] Vallières M., Rivest E.K., Perrin L.J., Liem X., Furstoss C., Aerts H.J.W.L., Khaouam N., Tan P.F.N., Wang C.H., Sultanem K. (2017). Radiomics strategies for risk assessment of tumour failure in head-and-neck cancer. Sci. Rep..

[55] Fidler I.J. (1990). Critical factors in the biology of human cancer metastasis: Twenty-eighth G.H.A. Clowes memorial award lecture. Cancer Res..

[56] Yokota J. (2000). Tumor progression and metastasis. Carcinogenesis.

[57] Granzier R.W.Y., Verbakel N.M.H., Ibrahim A., van Timmeren J.E., van Nijnatten T.J.A., Leijenaar R.T.H., Lobbes M.B.I., Smidt M.L., Woodruff H.C. (2020). MRI-based radiomics in breast cancer: Feature robustness with respect to inter-observer segmentation variability. Sci. Rep..

[58] Nikolov S., Blackwell S., Zverovitch A., Mendes R., Livne M., de Fauw J., Patel Y., Meyer C., Askham H., Paredes B.R. (2018). Deep learning to achieve clinically applicable segmentation of head and neck anatomy for radiotherapy. arXiv.

