# Supplementary material for: A Prospectively Validated Prognostic Model for Patients with Locally Advanced Squamous Cell Carcinoma of the Head and Neck Based on Radiomics of Computed Tomography Images
Source: Cancers (Basel). 2021 Jun 29;13(13):3271. doi: 10.3390/cancers13133271 (PMC8269129; doi:10.3390/cancers13133271)
Supplement: Supplementary file 1 [file cancers-13-03271-s001.zip › cancers-1230314-supplementary.pdf]

cancers

MDPI

Supplementary Materials

A Prospectively Validated Prognostic Model for Patients with Locally Advanced Squamous Cell Carcinoma of the Head and Neck Based on Radiomics of Computed Tomography Images

Simon A. Keek [1], Frederik W.R. Wesseling [2], Henry C. Woodruff [1,3], Janita E. van Timmeren [4], Irene H. Nauta [5], Thomas K. Hoffmann [6], Stefano Cavalieri [7], Giuseppina Calareso [8], Sergey Primakov [1], Ralph T. H. Leijenaar [9], Lisa Licitra [7,10], Marco Ravanelli [11], Kathrin Scheckenbach [12], Tito Poli [13], Davide Lanfranco [13], Marije R. Vergeer [14], C. René Leemans [5], Ruud H. Brakenhoff [5], Frank J.P. Hoebers [2] and Philippe Lambin [1,3,*]

[1] The D-Lab, Department of Precision Medicine, GROW-School for Oncology, Maastricht University, Maastricht, Universiteitssingel 40, 6229 ER Maastricht, The Netherlands; s.keek@maastrichtuniversity.nl (S.A.K.); h.woodruff@maastrichtuniversity.nl (H.C.W.); S.primakov@maastrichtuniversity.nl (S.P.P.)
[2] Department of Radiation Oncology (MAASTRO), GROW-School for Oncology and Developmental Biology, Maastricht University Medical Centre+, Postbus 3035, 6202 NA Maastricht, The Netherlands; frederik.wesseling@maastro.nl (F.W.R.W.); frank.hoebers@maastro.nl (F.J.P.H.)
[3] Department of Radiology and Nuclear Medicine, GROW-School for Oncology, Maastricht University Medical Centre+, P.O. Box 5800, 6202 AZ Maastricht, The Netherlands
[4] Department of Radiation Oncology, University Hospital Zürich, University of Zürich, Rämistrasse 100, 8091 Zürich, Switzerland; Janita.vanTimmeren@usz.ch
[5] Amsterdam UMC, Otolaryngology/Head and Neck Surgery, Cancer Center Amsterdam, Vrije Universiteit Amsterdam, Postbus 7057, 1007 MB Amsterdam, The Netherlands; i.nauta@amsterdamumc.nl (I.H.N.); cr.leemans@amsterdamumc.nl (C.R.L.); rh.brakenhoff@amsterdamumc.nl (R.H.B.)
[6] Department of Otorhinolaryngology, Head Neck Surgery, i2SOUL Consortium, University of Ulm, Frauensteige 14a (Haus 18), 89075 Ulm, Germany; t.hoffmann@uniklinik-ulm.de
[7] Head and Neck Medical Oncology Unit, Fondazione IRCCS Istituto Nazionale dei Tumori, via Giacomo Venezian, University of Milan, 1 20133 Milano, Italy; stefano.cavalieri@istitutotumori.mi.it (S.C.); lisa.licitra@istitutotumori.mi.it (L.L.)
[8] Radiology Unit, Fondazione IRCCS Istituto Nazionale dei Tumori via Giacomo Venezian, 1 20133 Milano, Italy; giuseppina.calareso@istitutotumori.mi.it
[9] Oncoradiomics SA, Liège, Clos Chanmurly 13, 4000 Liège, Belgium; ralph.leijenaar@oncoradiomics.com
[10] Department of Oncology and Hemato-Oncology, University of Milan, Via S. Sofia 9/1, 20122 Milano, Italy
[11] Department of Medicine and Surgery, University of Brescia, Viale Europa, 11-25123 Brescia, Italy; marcoravanelli@hotmail.it
[12] Department. of Otorhinolaryngology-Head and Neck Surgery, University Hospital Düsseldorf, Moorenstr. 5, 40225 Düsseldorf, Germany; Scheckenbach@med.uni-duesseldorf.de
[13] Maxillofacial Surgery Unit, Department of Medicine and Surgery, University of Parma-University Hospital of Parma, via Università, 12-I, 43121 Parma, Italy; tito.poli@unipr.it (T.P.); lanfranco82@yahoo.it (D.L.)
[14] Amsterdam UMC, Cancer Center Amsterdam, Department of Radiation Oncology, Vrije Universiteit Amsterdam, Postbus 7057, 1007 MB Amsterdam, The Netherlands; mr.vergeer@amsterdamumc.nl
* Correspondence: philippe.lambin@maastrichtuniversity.nl; Tel.: +32 475 259596

Supplementary Materials

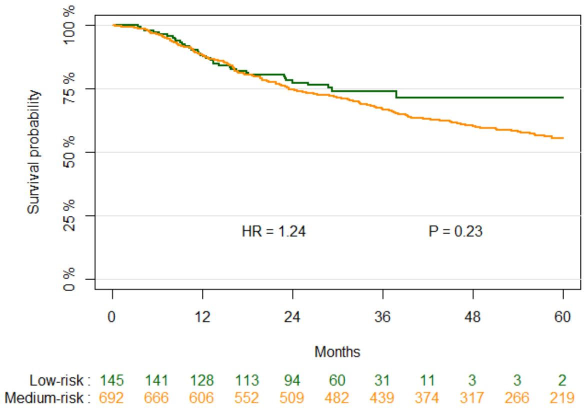

Figure S1. Kaplan-Meier survival curves of the retrospective and prospective patient cohort. P-value of the log-rank test and hazard ratio between the two patient cohorts are displayed.

Table S1: Treatment characteristics of the entire patient cohort.

**Center**		**Chemotherapy (% / N patients)**	**Radiotherapy (% / N patients)**	**Surgery (% / N patients)**
AOP		10 / 7	64 / 43	100 / 67
Brescia		0 / 0	0 / 0	80 / 4
INT		55 / 37	87 / 58	63 / 42
Maastro		43 / 115	100 / 265	15 / 40
UDUS		55 / 47	81 / 69	71 / 60
Ulm		69 / 11	100 / 16	88 / 14
VUmc		63 / 190	100 / 304	23 / 69
Stage 7th edition	III	32 / 78	95 / 234	31 / 77
	IVA	58 / 282	92 / 446	40 / 196
	IVB	61 / 47	97 / 75	20 / 23
Tumor location	Hypopharynx	66 / 77	98 / 115	32 / 37
	Larynx	31 / 64	95 / 199	34 / 72
	Oral Cavity	30 / 43	78 / 110	80 / 113
	Oropharynx	66 / 223	97 / 331	22 / 74

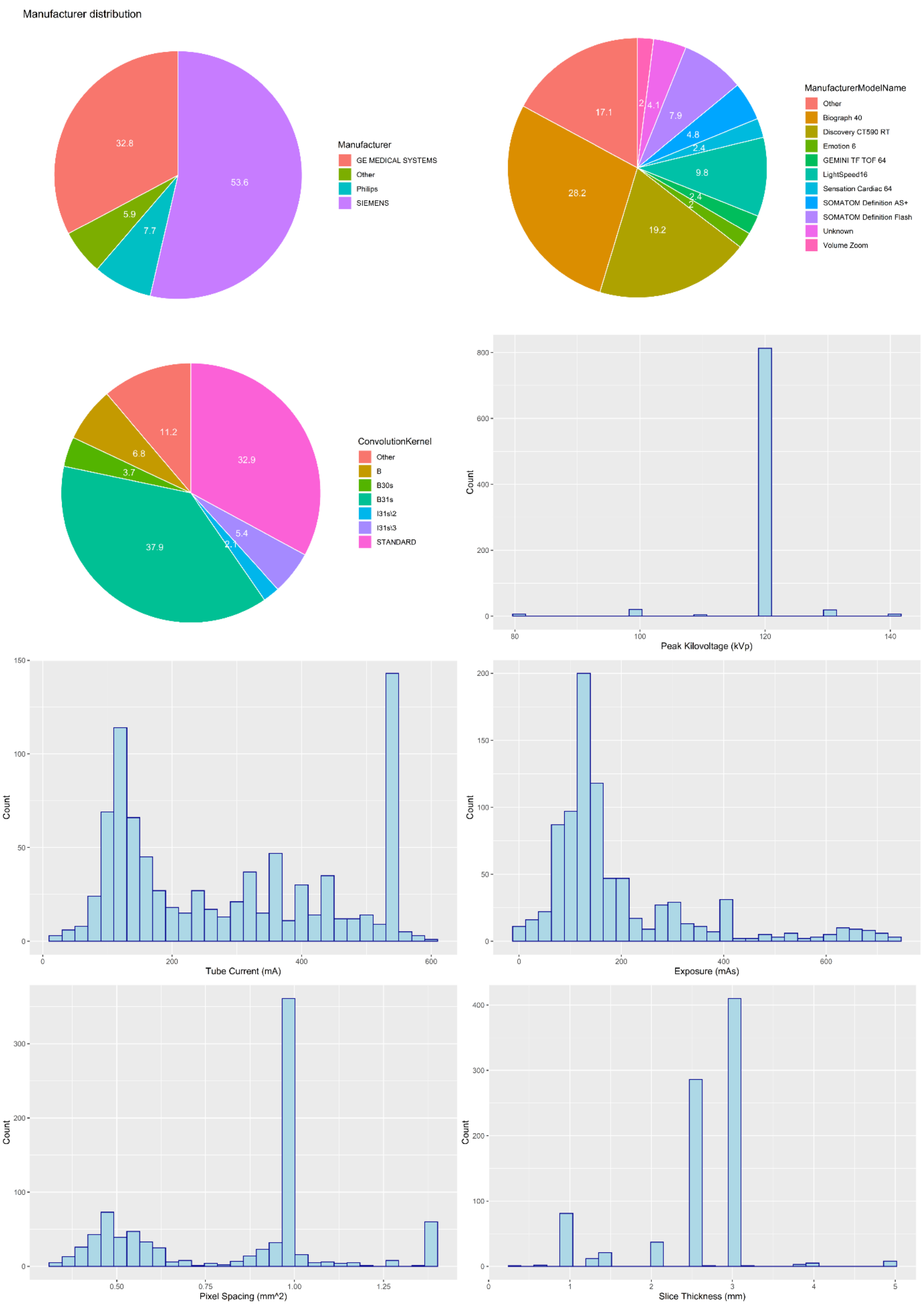

Figure S2. Distributions of imaging acquisition parameters for the full patient population (N=809).

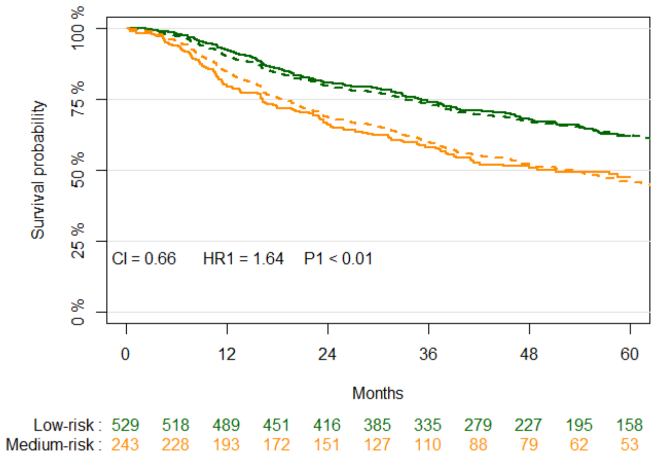

Figure S3. Kaplan-Meier survival cohorts of the full patient cohort (N=772) stratified based on the previously created signature, showing the p-value of the split between risk-groups, model performance through the CI and the HR between the risk groups. The solid lines represent the observed survival curves, the dashed the corresponding predicted survival curves.

Figure S4 shows Kaplan-Meier survival curves for the prospective cohort after stratification based on tumor volume, with a CI of 0.68. The p-values of the log-rank test of the low and medium and medium and high split were 0.62 and <0.01, respectively.

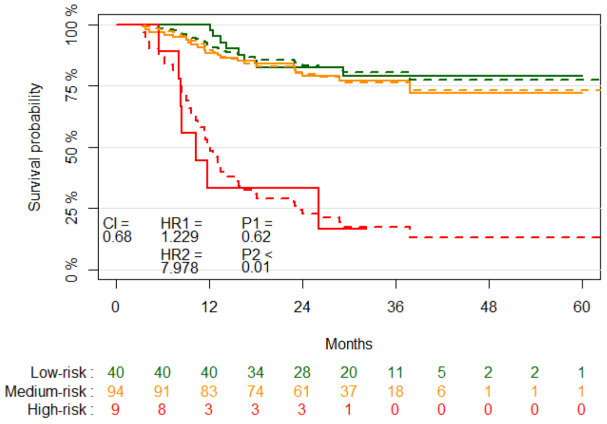

Figure S4. Kaplan-Meier survival cohorts of the prospective patient cohort (N=143) stratified based on tumor volume. P-value of the log-rank tests, CI of the model performance, and hazard ratios are displayed. The solid lines represent the observed survival curves, the dashed the corresponding predicted survival curves.

Figure S5 shows Kaplan-Meier survival curves for the prospective cohort after stratification based on TNM8, with a CI of 0.74. The P-values of the log-rank test of the low and

medium and medium and high split were both <0.01.

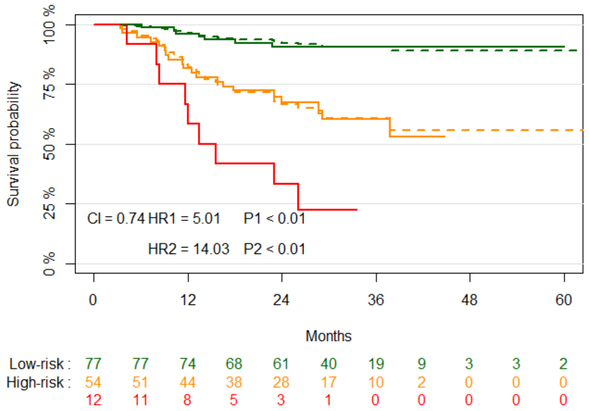

Figure S5. Kaplan-Meier survival curves of the prospective cohort (N=143) stratified based on TNM8.P-value of the log-rank tests, CI of the model performance, and hazard ratios are displayed. The solid lines represent the observed survival curves, the dashed the corresponding predicted survival curves.

Figure S6 shows Kaplan-Meier survival curves of the prospective cohort after stratification based on clinical and biological features, with a CI of 0.73 in validation. The p-value of the log-rank test of the low and medium split was <0.01, but the p-value of the log-rank test of the medium and high split was not significant at 0.57.

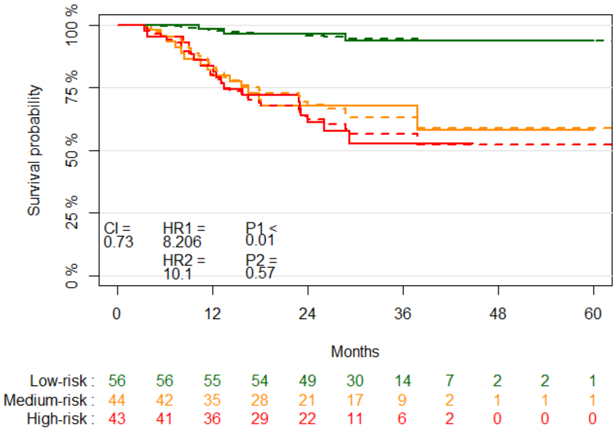

Figure S6. Kaplan-Meier survival cohorts of the prospective patient cohort (N=143) stratified based on clinical and biological parameters., P-value of the split between risk-groups, CI of the model performance, and hazard ratios are displayed. The solid lines represent the observed survival curves, the dashed the corresponding predicted survival curves.

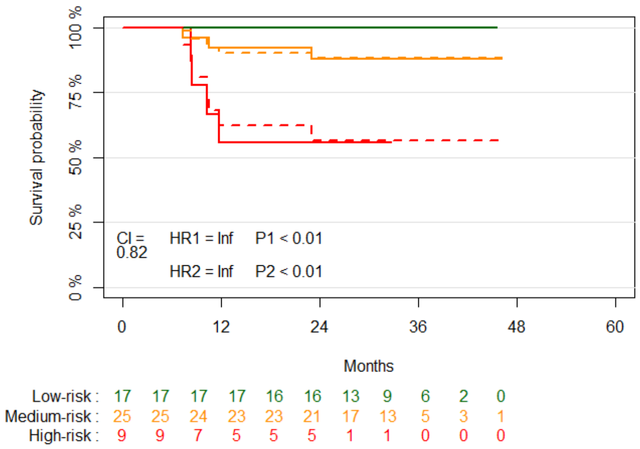

Figure S7. Kaplan-Meier survival curves of the oropharynx prospective patient (N=51) cohort using radiomics features, showing log-rank test p-value of the split between risk groups and the CI of the model-performance in the prospective cohort. Risk group split based on median training prediction value. The solid lines represent the observed survival curves, the dashed the corresponding predicted survival curves.

Ethical approval

The study procedures of the BD2Decide project were approved in accordance with the Declaration of Helsinki, the European and local ethical conventions and legal aspects, as well as the European General Data Protection Regulation. The management and exchange of data, specimens, and imaging information were regulated between the partners through data and material transfer agreements and standard operating procedures. Central data, imaging, and material were anonymized by the centers prior to aggregation, and data were stored in a secured and locked information technology surrounding according to General Data Protection Regulation (GDPR).

Data description and inclusion criteria

Patient data were acquired from seven different centers: Maastricht Radiation Oncology (MAASTRO), Amsterdam UMC, location VUmc, Heinrich-Heine-Universität Düsseldorf (UDUS), University Ulm (UU), Fondazione IRCCS Istituto Nazionale dei Tumori Milano (INT), Azienda Ospedaliero Universitaria di Parma (AOP), and University of Brescia (UB). The data collected included clinical, biological, pathological, and radiological variables for each case. Only patients age 18 years or above with HNSCC confirmed by histological examinations, a clinical TNM stage III, IVA, or IVB based on AJCC 7th edition, treated with curative intent (any combination of surgery, radiotherapy, and chemotherapy), that had pre-treatment tumor specimens contrast-enhanced CT scan of the head and neck region available were included.

Radiomic features description

Features can be can be divided into first-order HU intensity, histogram statistics, shape, and texture features. First order HU intensity and histogram statistics describe the total distribution of voxel intensities over the CT image. Shape features describe two- and three-dimensional size and shape of the GTV. Tumor volume measured through the voxel volume of the GTV is also a radiomics feature and can be seen as a more complex and complete feature than the size used for TNM staging. Texture features describe the relative spatial distribution of intensity values derived from 6 different matrices that are defined over the images: gray-level co-occurrence (GLCM)[1], gray-level run length

(GLRLM)[2], grey-level size-zone (GLSZM)[3], gray-level distance-zone (GLDZM)[4], gray-level dependence (NGLDM)[5], and neighborhood gray-tone difference matrix (NGTDM).[6] In addition, more images are created by applying two types of image filtering techniques to the original image. These extra filtered images are then used to extract the earlier described first-order, histogram, and texture features. The first technique is wavelet filtering, which involves 3D coif wavelet transforms along the three axes of the original images at 2 spatial frequencies (high and low) to decompose the images into 8 decomposed scans. The second filtering technique is Laplacian of Gaussian (LoG), which highlights regions of intensity change within an image The LoG-filter was applied with 4 different standard deviation values (2-5 mm) of the Gaussian filter, resulting in 4 different LoG-filtered images.

Pre- and post-processing

To make radiomics features rotationally invariant, and allow for features in different patient populations to be interchangeable, [7, 8], a 'sitkBSpline' interpolator was used to resample all images to uniform 1x1x3 mm3 voxel sizes. The choice for voxel dimensions was made based on majority ruling, where we found that most patients had a slice spacing of 3mm and pixel spacing of ~1mm. Furthermore, as differences in gray level intensity distributions also affect reproducibility and to make computation of features more efficient [7,9], the intensity values were set to a fixed 25 Hounsfield Units (HU) bin-size, resulting in images with ranges of 16-128 bins. This number of bins was chosen as a balance between reducing noise and limiting the size of the texture matrices on one hand and retaining a minimum contrast level in the lesions with less intensity ranges on the other. Disconnected voxels were removed to ensure only one fully connected structure was used for feature calculations. All radiomics features, besides shape features, had their Z-score normalization metrics (mean and scale) measured in the training dataset and applied to the features in both datasets. Any feature that failed to extract for any of the patients, for example because a filter was too large to apply to a smaller lesion, was removed. This strategy was adopted since all features selected for the signature need to be applicable to all (future) patients. Any feature with near-zero variance was also removed, as these features do not contain any useful information for a model.

Model calibration

The prognostic indices (PI), or linear predictors, of the training and validation dataset were determined. The PI is defined as $\sum_i x_i \beta_i$, which is the sum of the model's variables x multiplied by the regression coefficients β. To determine the calibration slope, Cox regression was performed on the PI, and the unity value of the slope was tested through a log-rank test. Afterwards, a joint log-rank test on all the predictors plus the offset of the PI was performed, and tested for non-significance, which would indicate a good fit for our model.

Clinical and biological covariates

The full list of clinical covariates was: age at diagnosis, sex, ACE-27 comorbidity score, smoking pack years, AJCC 8th edition TNM staging, smoking at time of diagnosis (yes/no/former, where former is defined as having stopped before enrolment), and alcohol consumption at time of diagnosis (yes/no/former, where former is defined as having stopped before enrolment). The list of biological covariates was: Hemoglobin (Hb) level, and HPV-status. P16 status was determined through p16 immunostaining. For p16 positive cases, this was followed by HPV DNA PCR confirmation, which determines HPV status. Patients which were found to be p16 positive but tested negative for HPV, were considered HPV negative.

Table S2. Table of used R packages.

Purposes	Functions	Packages	Versions
Spearman's rank correlation	'cor'	'stats'	3.6.3
ROC plots, AUC values, and test	'roc'	'pROC'	1.16.2
Feature selection	'nearZeroVar', 'uni.selection'	'caret', 'compound.cox'	6.0-86, 3.19
Cox proportional hazard modelling	'coxph', 'Surv'	'survival'	3.1.12
Harrel's C-index	'rcorr.cens'	'Hmisc'	4.4.0
Cox Survival Estimates	'survest'	'rms'	5.1.4
Create survival curves	'survfit'	'survest'	3.1.12
Drawing survival curves	'ggsurvplot'	'survminer'	0.4.7
Missing value imputation	'missForest'	'missForest'	1.4

Table S3. Selected radiomics features for the retrospective training cohort.

#	Name feature
1	log.sigma.5.0.mm.3D_glszm_GrayLevelNonUniformity
2	wavelet.HLH_glszm_ZoneEntropy
3	wavelet.HLL_glszm_ZoneEntropy
4	wavelet.LLH_glszm_ZoneEntropy
5	original_shape_Sphericity
6	log.sigma.4.0.mm.3D_gldm_DependenceEntropy
7	wavelet.HHH_glrlm_LowGrayLevelRunEmphasis
8	wavelet.HHL_glszm_ZoneEntropy
9	log.sigma.5.0.mm.3D_gldm_LowGrayLevelEmphasis
10	original_firstorder_Kurtosis
11	log.sigma.2.0.mm.3D_glrlm_RunEntropy

Table S4. Selected radiomics features for the retrospective oropharynx training cohort.

#	Name feature
1	original_shape_MajorAxisLength
2	wavelet.HHL_glszm_GrayLevelNonUniformity
3	log.sigma.5.0.mm.3D_glszm_GrayLevelNonUniformity
4	original_shape_Sphericity
5	wavelet.LLH_glszm_ZoneEntropy
6	original_firstorder_Maximum
7	log.sigma.4.0.mm.3D_glrlm_RunEntropy
8	wavelet.HLL_glszm_ZoneEntropy

RQS and TRIPOD

The radiomics quality score (RQS) assesses the validity of the overall radiomics workflow, and in particular the (external) validation. The RQS consists of 16 components, which together count up to a maximum of 36 points. Similarly, we followed the general procedure recommended in transparent reporting of a multivariable prediction model for individual prognosis or diagnosis (TRIPOD).[10] This guideline consists of a 22-point checklist that covers more general principles for articles including careful reporting and article structuring. The calculated RQS was 75%. A significant portion of points were lost in criterium 11, as we did not apply for a clinical trial to test the signature created in this study. An overview of the point allocation is shown in Table S5. For the TRIPOD statement, and adherence of 76% was calculated. An overview of the point allocation is shown in Table S6.

Table S5. Radiomics quality score checklist, as formulated in earlier work.[11] The table displays the different criteria, the maximum amount of points that can be acquired (or maximum points that can be deducted) and the points calculated in this study.

1	Image protocol quality - well-documented image protocols (for example, contrast, slice thickness, energy, etc.) and/or usage of public image protocols allow reproducibility/replicability	+ 1 (if protocols are well-documented) + 1 (if public protocol is used)	1
2	Multiple segmentations - possible actions are: segmentation by different physicians/algorithms/software, perturbing segmentations by (random) noise, segmentation at different breathing cycles. Analyse feature robustness to segmentation variabilities	1	1
3	Phantom study on all scanners - detect inter-scanner differences and vendor-dependent features. Analyse feature robustness to these sources of variability	1	0
4	Imaging at multiple time points - collect images of individuals at additional time points. Analyse feature robustness to temporal variabilities (for example, organ movement, organ expansion/shrinkage)	1	0
5	Feature reduction or adjustment for multiple testing - decreases the risk of overfitting. Overfitting is inevitable if the number of features exceeds the number of samples. Consider feature robustness when selecting features	− 3 (if neither measure is implemented) + 3 (if either measure is implemented)	3
6	Multivariable analysis with non radiomics features (for example, EGFR mutation) - is expected to provide a more holistic model. Permits correlating/inferencing between radiomics and non radiomics features	1	1
7	Detect and discuss biological correlates - demonstration of phenotypic differences (possibly associated with underlying gene–protein expression patterns) deepens understanding of radiomics and biology	1	1
8	Cut-off analyses - determine risk groups by either the median, a previously published cut-off or report a continuous risk variable. Reduces the risk of reporting overly optimistic results	1	1
9	Discrimination statistics - report discrimination statistics (for example, C-statistic, ROC curve, AUC) and their statistical significance (for example, p-values, confidence intervals). One can also apply resampling method (for example, bootstrapping, cross-validation)	+ 1 (if a discrimination statistic and its statistical significance are reported) + 1 (if a resampling method technique is also applied)	2
10	Calibration statistics - report calibration statistics (for example, Calibration-in-the-large/slope, calibration plots) and their statistical significance (for example, P-values, confidence intervals). One can also apply resampling method (for example, bootstrapping, cross-validation)	+ 1 (if a calibration statistic and its statistical significance are reported) + 1 (if a resampling method technique is also applied)	1
11	Prospective study registered in a trial database - provides the highest level of evidence	+ 7 (for prospective validation of a radiomics signature in an appropriate trial)	0

	supporting the clinical validity and usefulness of the radiomics biomarker		
12	Validation - the validation is performed without retraining and without adaptation of the cut-off value, provides crucial information with regard to credible clinical performance	- 5 (if validation is missing) + 2 (if validation is based on a dataset from the same institute) + 3 (if validation is based on a dataset from another institute) + 4 (if validation is based on two datasets from two distinct institutes) + 4 (if the study validates a previously published signature) + 5 (if validation is based on three or more datasets from distinct institutes)	9
13	Comparison to 'gold standard' - assess the extent to which the model agrees with/is superior to the current 'gold standard' method (for example, TNM-staging for survival prediction). This comparison shows the added value of radiomics	2	2
14	Potential clinical utility - report on the current and potential application of the model in a clinical setting (for example, decision curve analysis).	2	2
15	Cost-effectiveness analysis - report on the cost-effectiveness of the clinical application (for example, QALYs generated)	1	0
16	Open science and data - make code and data publicly available. Open science facilitates knowledge transfer and reproducibility of the study	+ 1 (if scans are open source) + 1 (if region of interest segmentations are open source) + 1 (if code is open source) + 1 (if radiomics features are calculated on a set of representative ROIs and the calculated features and representative ROIs are open source)	3
	Total score:	36	27

Table S6. TRIPOD statement checklist as defined in previous work[10], filled out for the present study.

	**Y=yes; N=no; R=referenced; NA=not applicable**	**Development [D]**	**External validation [V]**	**Combined Development & External validation [D+V]**
	**Title and abstract**			
**1**	**Identify the study as developing and/or validating a multivariable prediction model, the target population, and the outcome to be predicted.**			**0**
i	The words developing/development, validation/validating, incremental/added value (or synonyms) are reported in the title	N	N	N
ii	The words prediction, risk prediction, prediction model, risk models, prognostic models, prognostic indices, risk scores (or synonyms) are reported in the title	Y	Y	Y
iii	The target population is reported in the title	Y	Y	Y
iv	The outcome to be predicted is reported in the title	Y	Y	Y
**2**	**Provide a summary of objectives, study design, setting, participants, sample size, predictors, outcome, statistical analysis, results, and conclusions.**			**0**
i	The objectives are reported in the abstract	Y	Y	Y

ii	Sources of data are reported in the abstract<br>*E.g. Prospective cohort, registry data, RCT data.*	Y	Y	Y
iii	The setting is reported in the abstract<br>*E.g. Primary care, secondary care, general population, adult care, or paediatric care. The setting should be reported for both the development and validation datasets, if applicable.*	Y	Y	Y
iv	A general definition of the study participants is reported in the abstract<br>*E.g. patients with suspicion of certain disease, patients with a specific disease, or general eligibility criteria.*	Y	Y	Y
v	The overall sample size is reported in the abstract	Y	Y	Y
vi	The number of events (or % outcome together with overall sample size) is reported in the abstract<br>*If a continuous outcome was studied, score Not applicable (NA).*	N	N	N
vii	Predictors included in the final model are reported in the abstract. For validation studies of well-known models, at least the name/acronym of the validated model is reported<br>*Broad descriptions are sufficient, e.g. 'all information from patient history and physical examination'.*<br>*Check in the main text whether all predictors of the final model are indeed reported in the abstract.*	Y	Y	Y
viii	The outcome is reported in the abstract	Y	Y	Y
ix	Statistical methods are described in the abstract<br>*For model development, at least the type of statistical model should be reported. For validation studies a quote like "model's discrimination and calibration was assessed" is considered adequate. If done, methods of updating should be reported.*	Y	Y	Y
x	Results for model discrimination are reported in the abstract<br>*This should be reported separately for development and validation if a study includes both development and validation.*	Y	Y	Y
xi	Results for model calibration are reported in the abstract<br>*This should be reported separately for development and validation if a study includes both development and validation.*	N	N	N
xii	Conclusions are reported in the abstract<br>*In publications addressing both model development and validation, there is no need for separate conclusions for both; one conclusion is sufficient.*	Y	Y	Y
**3a**	**Explain the medical context (including whether diagnostic or prognostic) and rationale for developing or validating the multivariable prediction model, including references to existing models.**			**1**
i	The background and rationale are presented	Y	Y	Y
ii	Reference to existing models is included (or stated that there are no existing models)	Y	Y	Y
**3b**	**Specify the objectives, including whether the study describes the development or validation of the model or both.**			**1**
i	It is stated whether the study describes development and/or validation and/or incremental (added) value	Y	Y	Y
	**Methods**			
**4a**	**Describe the study design or source of data (e.g., randomized trial, cohort, or registry data), separately**			**1**

	**for the development and validation data sets, if applicable.**			
i	The study design/source of data is described<br>*E.g. Prospectively designed, existing cohort, existing RCT, registry/medical records, case control, case series.*<br>*This needs to be explicitly reported; reference to this information in another article alone is insufficient.*	Y	Y	Y
**4b**	**Specify the key study dates, including start of accrual; end of accrual; and, if applicable, end of follow-up.**			**1**
i	The starting date of accrual is reported	Y	Y	Y
ii	The end date of accrual is reported	Y	Y	Y
iii	The length of follow-up and prediction horizon/time frame are reported, if applicable<br>*E.g. "Patients were followed from baseline for 10 years" and "10-year prediction of…"; notably for prognostic studies with long term follow-up.*<br>*If this is not applicable for an article (i.e. diagnostic study or no follow-up), then score Not applicable (NA).*	Y	Y	Y
**5a**	**Specify key elements of the study setting (e.g., primary care, secondary care, general population) including number and location of centres.**			**1**
i	The study setting is reported (e.g. primary care, secondary care, general population)<br>*E.g.: 'surgery for endometrial cancer patients' is considered to be enough information about the study setting.*	Y	Y	Y
ii	The number of centres involved is reported<br>*If the number is not reported explicitly, but can be concluded from the name of the centre/centres, or if clearly a single centre study, score Yes.*	Y	Y	Y
iii	The geographical location (at least country) of centres involved is reported<br>*If no geographical location is specified, but the location can be concluded from the name of the centre(s), score Yes.*	Y	Y	Y
**5b**	**Describe eligibility criteria for participants.**			**1**
i	In-/exclusion criteria are stated<br>*These should explicitly be stated. Reasons for exclusion only described in a patient flow is not sufficient.*	Y	Y	Y
**5c**	**Give details of treatments received, if relevant.**<br>*(i.e. notably for prognostic studies with long term follow-up)*			**1**
i	Details of any treatments received are described<br>*This item is notably for prognostic modelling studies and is about treatment at baseline or during follow-up. The 'if relevant' judgment of treatment requires clinical knowledge and interpretation.*<br>*If you are certain that treatment was not relevant, e.g. in some diagnostic model studies, score Not applicable.*	Y	Y	Y
**6a**	**Clearly define the outcome that is predicted by the prediction model, including how and when assessed.**			**1**
i	The outcome definition is clearly presented<br>*This should be reported separately for development and validation if a publication includes both.*	Y	Y	Y
ii	It is described how outcome was assessed (including all elements of any composite, for example CVD [e.g. MI, HF, stroke]).	Y	Y	Y
iii	It is described when the outcome was assessed (time point(s) since T0)	Y	Y	Y

**6b**	**Report any actions to blind assessment of the outcome to be predicted.**			**0**
i	Actions to blind assessment of outcome to be predicted are reported<br>*If it is clearly a non-issue (e.g. all-cause mortality or an outcome not requiring interpretation), score Yes. In all other instances, an explicit mention is expected.*	N	N	N
**7a**	**Clearly define all predictors used in developing or validating the multivariable prediction model, including how and when they were measured.**			**1**
i	All predictors are reported<br>*For development, "all predictors" refers to all predictors that potentially could have been included in the 'final' model (including those considered in any univariable analyses). For validation, "all predictors" means the predictors in the model being evaluated.*	Y	Y	Y
ii	Predictor definitions are clearly presented	Y	Y	Y
iii	It is clearly described how the predictors were measured	Y	Y	Y
iv	It is clearly described when the predictors were measured	Y	Y	Y
**7b**	**Report any actions to blind assessment of predictors for the outcome and other predictors.**			**0**
i	It is clearly described whether predictor assessments were blinded for outcome<br>*For predictors for which it is clearly a non-issue (e.g. automatic blood pressure measurement, age, sex) and for instances where the predictors were clearly assessed before outcome assessment, score Yes. For all other predictors an explicit mention is expected.*	N	N	N
ii	It is clearly described whether predictor assessments were blinded for the other predictors	N	N	N
**8**	**Explain how the study size was arrived at.**			**1**
i	It is explained how the study size was arrived at<br>*Is there any mention of sample size, e.g. whether this was done on statistical grounds or practical/logistical grounds (e.g. an existing study cohort or data set of a RCT was used)?*	Y	Y	Y
**9**	**Describe how missing data were handled (e.g., complete-case analysis, single imputation, multiple imputation) with details of any imputation method.**			**1**
i	The method for handling missing data (predictors and outcome) is mentioned<br>*E.g. Complete case (explicit mention that individuals with missing values have been excluded), single imputation, multiple imputation, mean/median imputation.*<br>*If there is no missing data, there should be an explicit mention that there is no missing data for all predictors and outcome. If so, score Yes.*<br>*If it is unclear whether there is missing data (from e.g. the reported methods or results), score No.*<br>*If it is clear there is missing data, but the method for handling missing data is unclear, score No.*	Y	Y	Y
ii	If missing data were imputed, details of the software used are given<br>*When under 9i explicit mentioning of no missing data, complete case analysis or no imputation applied, score Not applicable.*	Y	Y	Y

iii	If missing data were imputed, a description of which variables were included in the imputation procedure is given<br>*When under 9i explicit mentioning of no missing data, complete case analysis or no imputation applied, score Not applicable.*	Y	Y	Y
iv	If multiple imputation was used, the number of imputations is reported<br>*When under 9i explicit mentioning of no missing data, complete case analysis or no imputation applied, score Not applicable.*	Y	Y	Y
**10a**	**Describe how predictors were handled in the analyses.**			**1**
i	For continuous predictors it is described whether they were modelled as linear, nonlinear (type of transformation specified) or categorized<br>*A general statement is sufficient, no need to describe this for each predictor separately.*<br>*If no continuous predictors were reported, score Not applicable.*	NA	Not applicable	NA
ii	For categorical or categorized predictors, the cut-points were reported<br>*If no categorical or categorized predictors were reported, score Not applicable.*	NA	Not applicable	NA
iii	For categorized predictors the method to choose the cut-points was clearly described<br>*If no categorized predictors, score Not applicable.*	NA	Not applicable	NA
**10b**	**Specify type of model, all model-building procedures (including any predictor selection), and method for internal validation.**			**0**
i	The type of statistical model is reported<br>*E.g. Logistic, Cox, other regression model (e.g. Weibull, ordinal), other statistical modelling (e.g. neural network)*	Y	Not applicable	Y
ii	The approach used for predictor selection <u>before</u> modelling is described<br>*'Before modelling' means before any univariable or multivariable analysis of predictor-outcome associations.*<br>*If no predictor selection before modelling is done, score Not applicable.*<br>*If it is unclear whether predictor selection before modelling is done, score No.*<br>*If it is clear there was predictor selection before modelling but the method was not described, score No.*	Y	Not applicable	Y
iii	The approach used for predictor selection <u>during</u> modelling is described<br>*E.g. Univariable analysis, stepwise selection, bootstrap, Lasso.*<br>*'During modelling' includes both univariable or multivariable analysis of predictor-outcome associations.*<br>*If no predictor selection during modelling is done (so-called full model approach), score Not applicable.*<br>*If it is unclear whether predictor selection during modelling is done, score No.*<br>*If it is clear there was predictor selection during modelling but the method was not described, score No.*	Y	Not applicable	Y
iv	Testing of interaction terms is described<br>*If it is explicitly mentioned that interaction terms were not addressed in the prediction model, score Yes.*	N	Not applicable	N

	If interaction terms were included in the prediction model, but the testing is not described, score No.			
v	Testing of the proportionality of hazards in survival models is described<br>*If no proportional hazard model is used, score Not applicable.*	Y	Not applicable	Y
vi	Internal validation is reported<br>*E.g. Bootstrapping, cross validation, split sample.*<br>*If the use of internal validation is clearly a non-issue (e.g. in case of very large data sets), score Yes. For all other situations an explicit mention is expected.*	Y	Not applicable	Y
**10c**	**For validation, describe how the predictions were calculated.**			**1**
i.	It is described how predictions for individuals (in the validation set) were obtained from the model being validated<br>*E.g. Using the original reported model coefficients with or without the intercept, and/or using updated or refitted model coefficients, or using a nomogram, spreadsheet or web calculator.*	Not applicable	Y	Y
**10d**	**Specify all measures used to assess model performance and, if relevant, to compare multiple models.**<br>*These should be described in methods section of the paper (item 16 addresses the reporting of the results for model performance).*			**1**
i	Measures for model discrimination are described<br>*E.g. C-index / area under the ROC curve.*	Y	Y	Y
ii	Measures for model calibration are described<br>*E.g. calibration plot, calibration slope or intercept, calibration table, Hosmer Lemeshow test, O/E ratio.*	Y	Y	Y
iii	Other performance measures are described<br>*E.g. R2, Brier score, predictive values, sensitivity, specificity, AUC difference, decision curve analysis, net reclassification improvement, integrated discrimination improvement, AIC.*	Y	Y	Y
**10e**	**Describe any model updating (e.g., recalibration) arising from the validation, if done.**			**Not applicable**
i	A description of model-updating is given<br>*E.g. Intercept recalibration, regression coefficient recalibration, refitting the whole model, adding a new predictor*<br>*If updating was done, it should be clear which updating method was applied to score Yes.*<br>*If it is not explicitly mentioned that updating was applied in the study, score this item as 'Not applicable'.*	Not applicable	NA	NA
**11**	**Provide details on how risk groups were created, if done.**<br>*If risk groups were not created, score this item as Yes.*			**1**
i	If risk groups were created, risk group boundaries (risk thresholds) are specified<br>*Score this item separately for development and validation if a study includes both development and validation.*<br>*If risk groups were not created, score this item as not applicable.*	Y	Y	Y
**12**	**For validation, identify any differences from the development data in setting, eligibility criteria, outcome and predictors.**			**0**

i	Differences or similarities in <u>definitions</u> with the development study are described<br>*Mentioning of any differences in all four (setting, eligibility criteria, predictors and outcome) is required to score Yes. If it is explicitly mentioned that there were no differences in setting, eligibility criteria, predictors and outcomes, score Yes.*	Not applicable	N	N
	**Results**			
**13a**	**Describe the flow of participants through the study, including the number of participants with and without the outcome and, if applicable, a summary of the follow-up time. A diagram may be helpful.**			**1**
i	The flow of participants is reported	Y	Y	Y
ii	The number of participants with and without the outcome are reported<br>*If outcomes are continuous, score Not applicable.*	Y	Y	Y
iii	A summary of follow-up time is presented<br>*This notably applies to prognosis studies and diagnostic studies with follow-up as diagnostic outcome. If this is not applicable for an article (i.e. diagnostic study or no follow-up), then score Not applicable.*	Y	Y	Y
**13b**	**Describe the characteristics of the participants (basic demographics, clinical features, available predictors), including the number of participants with missing data for predictors and outcome.**			**1**
i	Basic demographics are reported	Y	Y	Y
ii	Summary information is provided for all predictors included in the final developed/validated model	Y	Y	Y
iii	The number of participants with missing data for predictors is reported	Y	Y	Y
iv	The number of participants with missing data for the outcome is reported	Y	Y	Y
**13c**	**For validation, show a comparison with the development data of the distribution of important variables (demographics, predictors and outcome).**			**1**
i	Demographic characteristics (at least age and gender) of the validation study participants are reported along with those of the original development study	Not applicable	Y	Y
ii	Distributions of predictors in the model of the validation study participants are reported along with those of the original development study	Not applicable	Y	Y
iii	Outcomes of the validation study participants are reported along with those of the original development study	Not applicable	Y	Y
**14a**	**Specify the number of participants and outcome events in each analysis.**			**1**
i	The number of participants in each analysis (e.g. in the analysis of each model if more than one model is developed) is specified	Y	Not applicable	Y
ii	The number of outcome events in each analysis is specified (e.g. in the analysis of each model if more than one model is developed)<br>*If outcomes are continuous, score Not applicable.*	Y	Not applicable	Y
**14b**	**If done, report the unadjusted association between each candidate predictor and outcome.**			**0**
i	The unadjusted associations between each predictor and outcome are reported	N	Not applicable	N

	If any univariable analysis is mentioned in the methods but not in the results, score No. *If nothing on univariable analysis (in methods or results) is reported, score this item as Not applicable.*			
**15a**	**Present the full prediction model to allow predictions for individuals (i.e., all regression coefficients, and model intercept or baseline survival at a given time point).**			**1**
i	The regression coefficient (or a derivative such as hazard ratio, odds ratio, risk ratio) for each predictor in the model is reported	Y	Not applicable	Y
ii	The intercept or the cumulative baseline hazard (or baseline survival) for at least one time point is reported	Y	Not applicable	Y
**15b**	**Explain how to use the prediction model.**			**0**
i	An explanation (e.g. a simplified scoring rule, chart, nomogram of the model, reference to online calculator, or worked example) is provided to explain how to use the model for individualised predictions.	N	Not applicable	N
**16**	**Report performance measures (with confidence intervals) for the prediction model.** *These should be described in results section of the paper (item 10 addresses the reporting of the methods for model performance).*			**1**
i	A discrimination measure is presented *E.g. C-index / area under the ROC curve.*	Y	Y	Y
ii	The confidence interval (or standard error) of the discrimination measure is presented	Y	Y	Y
iii	Measures for model calibration are described *E.g. calibration plot, calibration slope or intercept, calibration table, Hosmer Lemeshow test, O/E ratio.*	Y	Y	Y
iv	Other model performance measures are presented *E.g. R2, Brier score, predictive values, sensitivity, specificity, AUC difference, decision curve analysis, net reclassification improvement, integrated discrimination improvement, AIC.*	Y	Y	Y
**17**	**If done, report the results from any model updating (i.e., model specification, model performance, recalibration).** *If updating was not done, score this TRIPOD item as 'Not applicable'.*			**Not applicable**
0	Model updating was done *If "No", then answer 17i-17v with "Not applicable"*	Not applicable	N	N
i	The updated regression coefficients for each predictor in the model are reported *If model updating was described as 'not needed', score Yes.*	Not applicable	NA	NA
ii	The updated intercept or cumulative baseline hazard or baseline survival (for at least one time point) is reported *If model updating was described as 'not needed', score Yes.*	Not applicable	NA	NA
iii	The discrimination of the updated model is reported	Not applicable	NA	NA
iv	The confidence interval (or standard error) of the discrimination measure of the updated model is reported	Not applicable	NA	NA
v	The calibration of the updated model is reported	Not applicable	NA	NA
	**Discussion**			

**18**	**Discuss any limitations of the study (such as nonrepresentative sample, few events per predictor, missing data).**			**1**
i	Limitations of the study are discussed<br>*Stating any limitation is sufficient.*	Y	Y	Y
**19a**	**For validation, discuss the results with reference to performance in the development data, and any other validation data.**			**1**
i	Comparison of results to reported performance in development studies and/or other validation studies is given	Not applicable	Y	Y
**19b**	**Give an overall interpretation of the results considering objectives, limitations, results from similar studies and other relevant evidence.**			**1**
i	An overall interpretation of the results is given	Y	Y	Y
**20**	**Discuss the potential clinical use of the model and implications for future research.**			**1**
i	The potential clinical use is discussed<br>*E.g. an explicit description of the context in which the prediction model is to be used (e.g. to identify high risk groups to help direct treatment, or to triage patients for referral to subsequent care).*	Y	Y	Y
ii	Implications for future research are discussed<br>*E.g. a description of what the next stage of investigation of the prediction model should be, such as "We suggest further external validation".*	Y	Y	Y
	**Other information**			
**21**	**Provide information about the availability of supplementary resources, such as study protocol, web calculator, and data sets.**			
i	Information about supplementary resources is provided	Y	Y	Y
**22**	**Give the source of funding and the role of the funders for the present study.**			**1**
i	The source of funding is reported or there is explicit mention that there was no external funding involved	Y	Y	Y
ii	The role of funders is reported or there is explicit mention that there was no external funding	Y	Y	Y
	**Number of applicable TRIPOD items**			**34**
	**Number of TRIPOD items adhered**			**26**
	**OVERALL adherence to TRIPOD**			**76%**

Supplementary References

1. Haralick RM, Shanmugam K, Dinstein I. Textural Features for Image Classification. IEEE Transactions on Systems, Man, and Cybernetics. 1973;SMC-3(6):610-21.

2. Galloway MM. Texture analysis using grey level run lengths. 1974 July 01, 1974.

3. Thibault G, Fertil B, Navarro C, Pereira S, Lévy N, Sequeira J, et al. Texture Indexes and Gray Level Size Zone Matrix Application to Cell Nuclei Classification2009.

4. Thibault G, Angulo J, Meyer F, editors. Advanced statistical matrices for texture characterization: Application to DNA chromatin and microtubule network classification. 2011 18th IEEE International Conference on Image Processing; 2011 11-14 Sept. 2011.

5. Sun C, Wee WG. Neighboring gray level dependence matrix for texture classification. Computer Vision, Graphics, and Image Processing. 1983;23(3):341-52.

6. Amadasun M, King R. Textural features corresponding to textural properties. IEEE Transactions on Systems, Man, and Cybernetics. 1989;19(5):1264-74.

7. Shafiq-Ul-Hassan M, Zhang GG, Latifi K, Ullah G, Hunt DC, Balagurunathan Y, et al. Intrinsic dependencies of CT radiomic features on voxel size and number of gray levels. Med Phys. 2017;44(3):1050-62.

8. Zwanenburg A, Vallieres M, Abdalah MA, Aerts H, Andrearczyk V, Apte A, et al. The Image Biomarker Standardization Initiative: Standardized Quantitative Radiomics for High-Throughput Image-based Phenotyping. Radiology. 2020:191145.

9. Lu L, Lv W, Jiang J, Ma J, Feng Q, Rahmim A, et al. Robustness of Radiomic Features in [(11)C]Choline and [(18)F]FDG PET/CT Imaging of Nasopharyngeal Carcinoma: Impact of Segmentation and Discretization. Mol Imaging Biol. 2016;18(6):935-45.

10. Collins GS, Reitsma JB, Altman DG, Moons KG. Transparent Reporting of a multivariable prediction model for Individual Prognosis Or Diagnosis (TRIPOD). Ann Intern Med. 2015;162(10):735-6.

11. Lambin P, Leijenaar RTH, Deist TM, Peerlings J, de Jong EEC, van Timmeren J, et al. Radiomics: the bridge between medical imaging and personalized medicine. Nature Reviews Clinical Oncology. 2017;14(12):749-62.
